# Scalable human neuronal models of tauopathy producing endogenous seed-competent 4R tau

**DOI:** 10.1126/sciadv.aeg1445

**Published:** 2026-07-31

**Authors:** Eliona Tsefou, Sumi Bez, Timothy J. Y. Birkle, Martha Foiani, Naoto Watamura, Mathieu Bourdenx, Daria Gavriouchkina, Emir Turkes, Samuel Crawford, Rachel Coneys, Adrian M. Isaacs, Karen E. Duff

**Affiliations:** ^1^ UK Dementia Research Institute, University College London, London, UK.; ^2^ Department of Neurodegenerative Disease, UCL Queen Square Institute of Neurology, London, UK.

## Abstract

The accumulation of pathological four-repeat (4R) tau is central to several frontotemporal dementia (FTD) subtypes, but human neuronal models amenable to high-throughput screening of 4R tau–targeting therapies remain very limited. To address this, we developed induced pluripotent stem cell (iPSC)–derived i^3^Neuron (i^3^N) lines expressing >75% 4R tau, driven by FTD splice-shifting mutations (Ser^305^Asn; S305N or S305N/IVS10 + 3). These neurons develop hyperphosphorylated tau and demonstrate somatodendritic mislocalization. These i^3^N neurons develop endogenous seed-competent tau and present pentameric formyl thiophene acetic acid–(pFTAA)-positive tau assemblies after 28 days in culture. For scalable screening, we CRISPR-engineered an HiBiT luminescence tag at the endogenous *MAPT* locus into the S305N/IVS10 + 3 iPSC line, enabling precise quantification of tau levels and pharmacological responses. The model responded predictably to compounds affecting tau clearance, demonstrating its suitability for drug discovery. Overall, this i^3^N platform recapitulates key features of 4R tauopathy and provides a robust system to identify therapeutic modulators of pathological tau.

## Teaser

A scalable human neuronal model recapitulates 4R tauopathy and enables discovery of modulators of pathological tau.

## INTRODUCTION

Tauopathies are a heterogeneous group of neurodegenerative disorders defined by the accumulation of pathological forms of the microtubule-associated protein tau (MAPT) in the brain. These include progressive supranuclear palsy (PSP), corticobasal degeneration (CBD), Pick’s disease, and frontotemporal dementia with Parkinsonism linked to chromosome 17 (FTDP-MAPT) (*1*). In these diseases, tau aggregates into intracellular fibrils that interfere with neuronal function and viability (*2*, *3*). Tau exists in six isoforms in the adult human brain, produced by alternative splicing of exons 2, 3, and 10 of the *MAPT* gene (*4*). These isoforms differ in the number of N-terminal inserts and in the presence of either three (3R) or four (4R) microtubule-binding repeats (*5*). During fetal development, the human brain predominantly expresses 3R tau (*6*), but in the healthy adult brain, the ratio of 3R:4R tau is balanced (*7*–*9*). Disruption of this balance, particularly an excess of 4R tau, is associated with several diseases (*10*, *11*).

Human induced pluripotent stem cell (iPSC)–derived neurons are a valuable tool to study early mechanisms of neurodegenerative diseases. However, using such human neuronal models to recapitulate 4R tau expression has historically been difficult as they typically express predominantly 0N3R tau, reflecting fetal-like tau splicing (*12*–*16*). Until recently, most of the iPSC-derived neuronal models that were able to induce moderate expression of 4R tau required prolonged and complicated culturing conditions, or they relied on the insertion of multiple tau mutations (*17*–*22*). A recent line also expressed an exon 10-11 fusion, with the highly aggregation-prone Pro^301^Leu/Ser^320^Phe (P301L/S320F) double mutation (*22*). Alternative models that make endogenous pathological neuronal tau include directly converted fibroblasts (microRNA-induced neurons, miNs) (*23*) and three-dimensional organoid cultures from ReNcell VM cells with amyloid copathology (*24*).

The S305N mutation, located at the end of exon 10, causes excessive production of 4R tau and consequently leads to FTDP-MAPT (*25*–*27*). Postmortem brain tissue from Ser^305^Asn (S305N) carriers exhibits 4R tau–positive inclusions, including neurofibrillary tangles and ring-like structures surrounding neuronal nuclei, reflecting severe tau pathology (*28*, *29*). A recent study has shown that an isogenic set of iPSC lines derived from an S305N mutation carrier has increased 4R tau after three months in culture (*30*). In the current study, we use i^3^Neuronal (i^3^N) versions of the same isogenic S305N iPSC set. Building on insights from a targeted, humanized mouse model, where combining both S305N and intronic IVS10 + 3 FTDP-MAPT–related mutations led to the expression of 99% 4R MAPT (*31*), we aimed to determine whether a similar shift could be achieved in a second set of lines derived from a different healthy donor. CRISPR-Cas9 editing was used in the second iPSC line to introduce the S305N point mutation and the IVS10 + 3 intronic mutation to further enhance 4R levels, as well as a HiBiT tag to monitor tau levels. The IVS10 + 3 mutation is spliced out of the protein, and elevated 4R tau resulted in tau hyperphosphorylation, changes in the cytoskeleton and, remarkably, the accumulation of endogenous seed-competent tau. We have tested this line with tau-modulating drugs, and we anticipate that it can be developed into a platform for drug screening, targeting tau in a range of neurodegenerative diseases.

## RESULTS

### i^3^N neurons with an S305N mutation predominantly express 4R tau after 7 days of differentiation

**Fig. 1.**
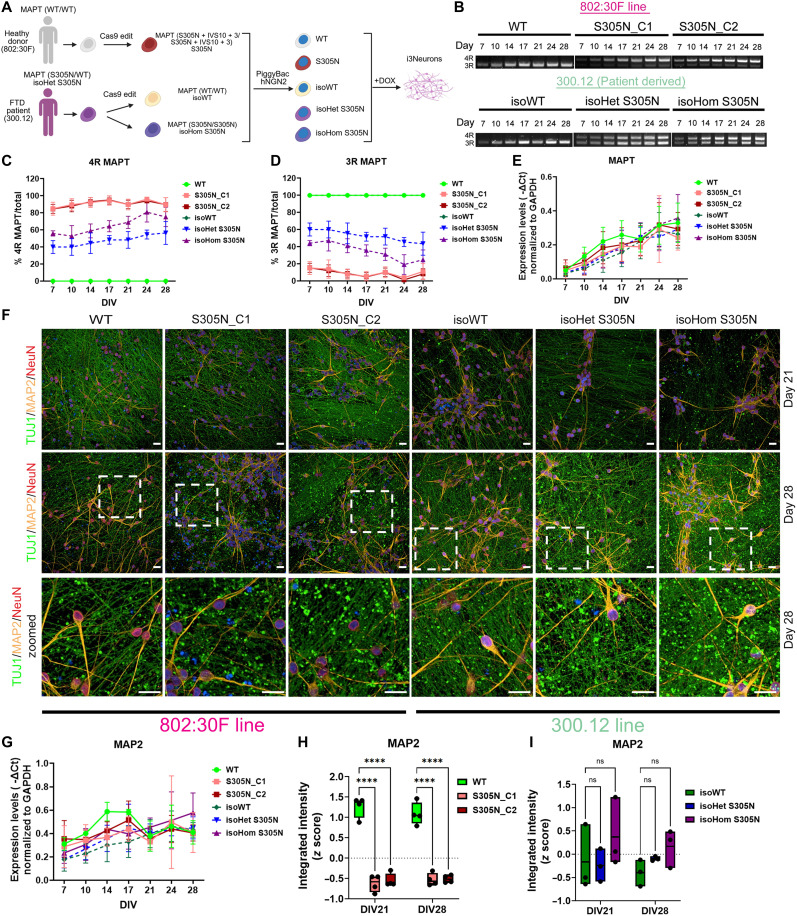
S305N i^3^N neurons express predominantly 4R tau. (**A**) Schematic representation of the iPSC lines used and the generation of iPSC-derived neurons via the piggyBac hNGN2 system, which enables neuronal differentiation [Created in BioRender. Tsefou, E. (2026) https://BioRender.com/80gh7nt]. (**B**) Representative semi-qPCR showing 3R and 4R tau isoform levels over time. (**C** and **D**) Quantification of 4R MAPT (C) and 3R MAPT (D) from (B) (mean ± SD, *n* = 4 to 7 independent differentiations per presented neuronal line). (**E**) Total MAPT mRNA levels measured by qPCR (means ± SD, *n* = 4 to 7 independent differentiations per presented neuronal line). (**F**) Representative images of methanol-fixed i3N neurons at DIV21 and DIV28 immunolabeled with TUJ1, MAP2, and NeuN. (**G**) MAP2 mRNA levels measured by qPCR (mean ± SD, *n* = 3 to 5 independent differentiations per presented neuronal line). Quantification of MAP2 expression from (F) in the 802:30F (**H**) and 300.12 (**I**) iPSC lines [two-way analysis of variance (ANOVA) followed by Tukey post hoc test; ****P < 0.0001; means ± SD, *n* = 3 to 4 independent differentiations per presented neuronal line]. ns, not significant.

The S305N mutation affects the RNA stem-loop structure that regulates alternative splicing of exon 10 in the *MAPT* gene (*32*). The IVS10 + 3 mutation lies adjacent to the splice-donor site and disrupts the stem-loop structure of the pre-mRNA, promoting increased exon 10 inclusion (*33*, *34*). To generate our model, we used a commercially available iPSC line from Synthego [802:30F, referred to as wild-type (WT)] and introduced the mutations using CRISPR-Cas9 with a single guide RNA (fig. S1A). As the IVS10 + 3 mutation is spliced out, only a single, FTDP-MAPT–causing mutation is present in the protein, and the cells are therefore a valid model of human 4R tauopathy. Two edited, homozygous clones, S305N_C1 and S305N_C2, were isolated and used for experiments. The top three off-target effects were validated by polymerase chain reaction (PCR) and sequencing, but no changes were observed. In addition to these lines, we used a patient-derived S305N heterozygous line (isoHet S305N), along with isogenic WT (isoWT) and homozygous (isoHom S305N) lines (Fig. 1A). These lines are available from National Centralized Repository for Alzheimer's Disease and Related Dementias (NCRAD) (*30*) and referred to in the current study as 300.12; for these lines, we only used one clone per genotype. All iPSC lines were transfected with a doxycycline-inducible human *Neurogenin-2* (hNGN2) transgene using the piggyBac transposon system (Fig. 1A) (*35*). Stable integration of hNGN2 enabled rapid differentiation into human excitatory cortical glutamatergic neurons (i^3^Ns) within 3 days of doxycycline exposure. Posttransfection, all lines maintained normal karyotypes (assessed via low-coverage whole genome sequencing) and continued to express pluripotency markers (fig. S1, B to E).

To assess the effect of the mutations on exon 10 inclusion, we differentiated all WT and mutant iPSC lines into i^3^N neurons using a two-step protocol adapted from Fernandopulle *et al.* (*36*), culturing them for up to 28 days. We examined MAPT isoform expression by semiquantitative PCR (semi-qPCR), focusing on the inclusion (4R MAPT) or exclusion (3R MAPT) of exon 10 (Fig. 1B). As expected, both WT and isoWT lines exclusively expressed 3R tau throughout differentiation [7 days in vitro (DIV7) to DIV28)]. In contrast, both S305N_C1 and C2 clones expressed >80% 4R tau as early as DIV7, increasing to >90% by DIV28. The isoHet S305N line exhibited a 3R:4R ratio of approximately 60:40 at DIV7, shifting to a nearly 50:50% ratio by DIV28. The isoHom S305N line started with 45:55% 3R:4R tau at DIV7 and reached ∼25:75% 3R:4R by DIV28. This line therefore had lower 4R levels compared to the S305N_C1 and C2 clones (Fig. 1, C and D). The level of total MAPT mRNA was quantified by reverse transcription qPCR and no change was observed (Fig. 1E). MAPT mRNA could not be detected in the neuronal lines before DIV7.

### Characterization of S305N i^3^N neurons

To assess the effects of the S305N mutation and 4R tau elevation on neuronal maturation, we analyzed neuronal marker expression at DIV21 and DIV28 by immunocytochemistry. All i^3^N neuronal lines were stained for βIII-tubulin (TUJ1) (an axonal marker), MAP2 (a somatodendritic marker), and NeuN (a nuclear marker associated with neuronal maturity) (Fig. 1F). No difference in TUJ1 expression was observed between S305N_C1 and C2 and the WT control at either time point (fig. S2A). In the isoHet and isoHom S305N lines, TUJ1 expression remained comparable to the isoWT control (fig. S2B). While overall TUJ1 expression was unchanged, we noted the emergence of irregular, swollen axonal protrusions in all lines expressing the S305N mutation, particularly at DIV28. These structures may represent axonal blebbing, which is commonly associated with neuronal stress, injury, or degeneration (*37*, *38*). We quantified the number of blebs in the total TUJ1 area and observed an increase in S305N_C1, isoHet S305N, and isoHom S305N, especially at DIV28, compared to respective isogenic i^3^N neurons (fig. S2, C and D). MAP2 mRNA levels did not differ between lines but increased over time in all neuronal cultures, reaching a plateau after DIV21 (Fig. 1G). However, the level of MAP2 protein was significantly reduced in both S305N_C1 and C2 neurons (Fig. 1H), whereas no change was detected in the isoHet and isoHom S305N lines (Fig. 1I). The observed reduction in MAP2 protein in the S305N_C1 and C2 lines may point to cytoskeletal instability. NeuN staining revealed that more than 80% of cells in all lines were NeuN-positive, indicating a high proportion of mature neurons (fig. S1E).

We also examined the expression of VGLUT1, a marker of glutamatergic neurons. VGLUT1 mRNA levels decreased over time, reaching a plateau around DIV17 (fig. S2F). At the protein level, immunocytochemistry at DIV21 and DIV28 revealed a statistically significant reduction in VGLUT1 expression in the S305N_C1 and C2 lines, with C1 showing nearly a 50% decrease compared to WT (fig. S2, G and H). Both S305N_C1 and C2 presented similar changes; however, for some measurements, a stronger phenotype was evident in the S305N_C1 neurons. No difference was observed in the isoHet and isoHom S305N lines at the time points studied (fig. S2I), which may reflect the lower levels of 4R (or higher relative levels of 3R tau) in these lines.

Last, we characterized the isoform composition of tau in each neuronal genotype. Protein samples were dephosphorylated and analyzed by Western blot with Tau13 immunolabeling. As expected, WT and isoWT neurons expressed predominantly 0N3R tau at DIV21 and DIV28, consistent with tau splicing profiles in iPSC-derived neurons (fig. S3J). In contrast, S305N_C1 neurons expressed only 0N4R tau across both time points. isoHet neurons exhibited a 1:1 ratio of 0N3R to 0N4R tau, while isoHom neurons expressed 58% 0N4R tau at DIV28.

### Mislocalization and distribution of tau in neurons expressing S305N mutant isoforms

**Fig. 2.**
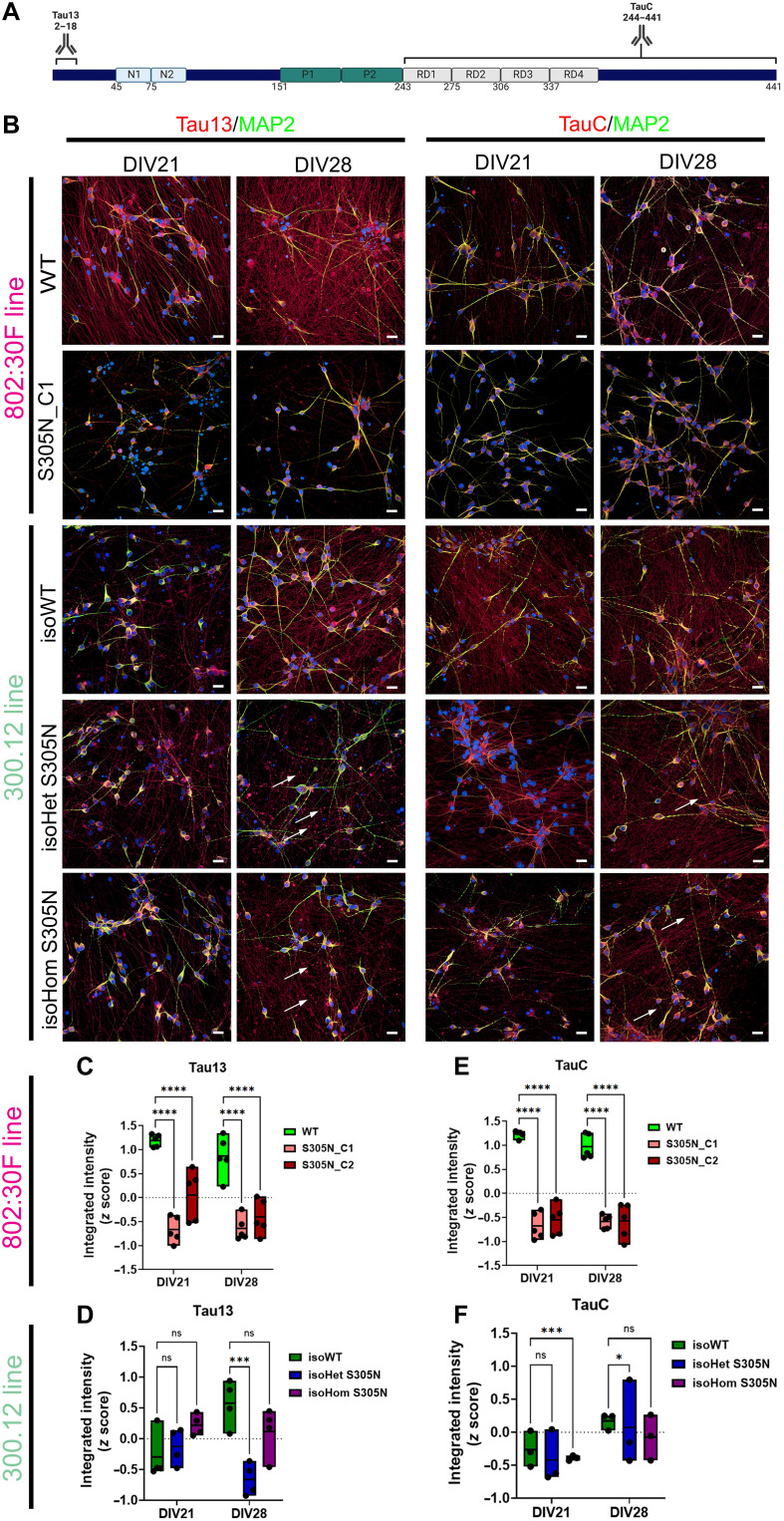
Distribution of total tau in S305N i^3^N neurons. (**A**) Schematic map of full-length tau and the epitope sites targeted by used total tau antibodies [Created in BioRender. Tsefou, E. (2026) https://BioRender.com/5sdt3za]. (**B**) Representative images of DIV21 and DIV28 i3N neurons double-immunolabeled with MAP2 and one of the total tau antibodies: Tau13 (left) and TauC (right). (**C** and **D**) Quantification of Tau13 staining in all imaged area (normalized to cell number), and (**E** to **F**) quantification of TauC staining in all imaged area (normalized to cell number) across all neuronal lines compared to their respective isogenic WT controls. [(C) to (F)] Data analyzed by two-way ANOVA followed by Tukey post-hoc test; means ± SD, *n* = 3 to 5 independent differentiations per presented neuronal line. Statistical significance: *P < 0.05, ***P < 0.001, ****P < 0.0001.

Tau undergoes proteolytic cleavage by various proteases, generating fragments that contribute to the pathogenesis of several neurodegenerative disorders (*39*, *40*). To investigate the subcellular distribution of tau, we performed immunocytochemistry on methanol-fixed neurons at DIV21 and DIV28 using antibodies targeting distinct tau domains: Tau13 (N terminus, epitopes 2 to 18 amino acids) and TauC (C terminus, epitopes 244 to 441 amino acids; Fig. 2A and fig. S3A for S305N_C2). In both WT and isoWT neurons, Tau13 and TauC labeled tau both in the somatodendritic compartment (colocalized with MAP2; Fig. 2B) and in the axons (fig. S3B, colocalized with TUJ1), with signal intensity increasing between DIV21 and DIV28 (Fig. 2B). S305N_C1 and C2 neurons displayed pronounced somatodendritic tau retention with markedly reduced axonal staining (Fig. 2B and fig. S3A and B). Densitometric analysis revealed an >80% reduction in total tau signal detected by Tau13 and TauC antibodies in this group (Fig. 2, C and E), with both clones presenting similar tau reduction. In the isoHet and isoHom S305N mutant neurons, tau remained detectable in both the somatodendritic compartment and axons (Fig. 2B and fig. S3B). Among the antibodies, Tau13 showed the least variability and revealed significantly reduced tau levels in isoHet neurons at DIV28, with a trend toward reduction observed in isoHom neurons (Fig. 2, D and F, 22% reduction, *P* = 0.0894).

Furthermore, neurons expressing isoHet and isoHom S305N tau displayed distinct tau-positive “blebs” at DIV28 when stained with Tau13 and TauC (Fig. 2B, arrows). Costaining with TUJ1 revealed partial colocalization of these tau blebs with tubulin-rich structures (fig. S3B, see arrows). Overall, these results suggest that high levels of 4R tau relative to 3R (or the effect of homozygous S305N mutation) led to both mislocalization and altered levels of tau, particularly affecting its axonal distribution. The most profound changes were observed in S305N_C1 and C2 neurons, where tau was almost exclusively confined to the somatodendritic compartment. We also quantified TauC levels within the MAP2-positive area, which followed a pattern similar to that observed across all compartments (fig. S3, C and D). The fact that both S305N_C1 and C2 showed a similar effect on total tau indicates that the observed results are not due to clonal selectivity or because of random integration of the hNGN2 cassette. While tau remained detectable in axons of isoHet and isoHom neurons, overall levels compared to isoWT trended lower, especially as revealed by Tau13 and TauC staining. Therefore, subsequent analyses were interpreted in the context of reduced total tau levels when possible.

### Increased tau phosphorylation in neurons expressing S305N mutant isoforms

**Fig. 3.**
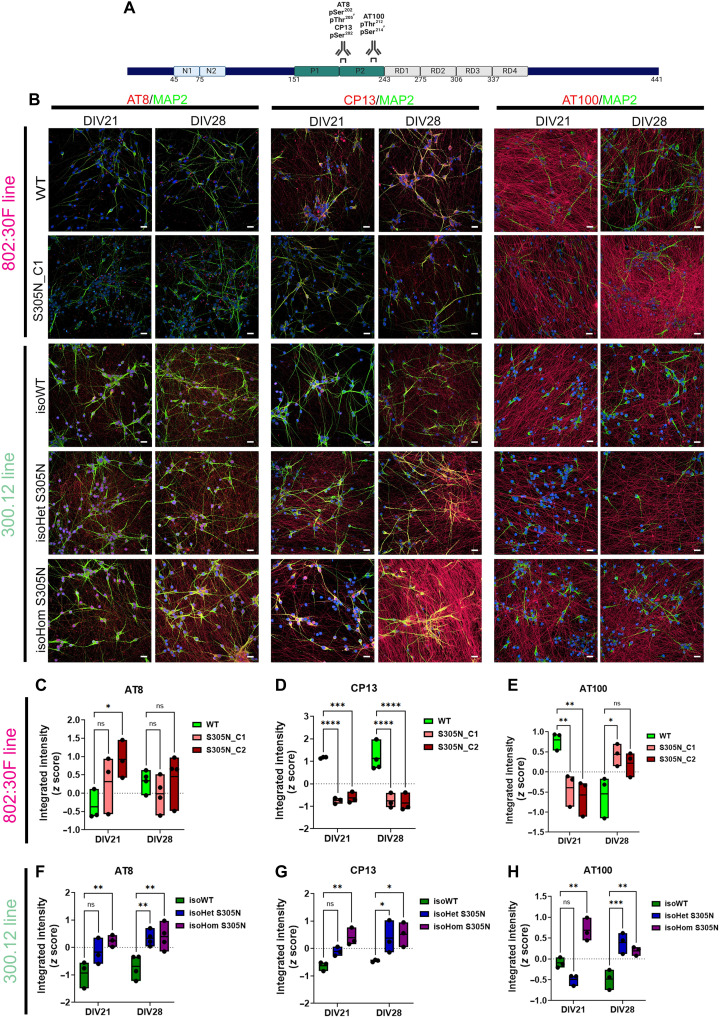
Distribution of pTau in S305N i^3^N neurons. (**A**) Schematic map of full-length tau and the epitope sites recognized by the pTau antibodies used [Created in BioRender. Tsefou, E. (2026) https://BioRender.com/5sdt3za]. (**B**) Representative images of DIV21 and DIV28 i^3^N neurons double-immunolabeled with MAP2 and one of the pTau antibodies: AT8 (left), CP13 (middle), and AT100 (right). (**C** to **H**) Quantification of AT8 (C and D), CP13 (E and F), and AT100 (G and H) staining across imaged area (normalized to cell number) in all neuronal lines compared to their respective isogenic WT controls. Statistical analysis was performed using two-way ANOVA followed by Tukey post hoc test, data are presented as means ± SD, *n* = 3 to 4 independent differentiations per presented neuronal line. Statistical significance: *P < 0.05, **P < 0.01, ***P < 0.001, ****P < 0.0001.

To investigate the effects of the S305N mutation on tau phosphorylation, we examined the relative levels and distribution of phosphorylated tau (pTau) epitopes using the following antibodies: AT8 (pSer^202^/pThr^205^), CP13 (pSer^202^), and AT100 (pThr^212^/pSer^214^). Immunocytochemistry was performed on methanol-fixed neurons at DIV21 and DIV28 (Fig. 3A and fig. S4A for S305N_C2). In S305N_C1 and C2 neurons, AT8 staining was predominantly localized to the somatodendritic compartment, whereas in the WT neurons AT8 staining was much lower (Fig. 3B). Quantitative analysis revealed a significant increase in AT8 signal at DIV21 in the S305N_C2 only (*P* = 0.025), whereas at DIV28, AT8 levels did not change (Fig. 3C). A similar trend was observed for S305N_C1, but that change did not reach significance. When normalizing AT8 levels to total tau (TauC), we observed a significant increase in the S305N_C1 only, at both time points (fig. S4C; AT8/TauC). CP13 staining in S305N_C1 and C2 neurons showed both somatodendritic and axonal localization. However, total CP13 signal was significantly reduced compared to WT (Fig. 3D). After normalization to TauC, a trend toward increased CP13 levels was observed at DIV28 (fig. S4E). Unexpectedly, AT100 staining in S305N_C1 and C2 neurons was restricted exclusively to the axons (Fig. 3B). Given the overall reduction in total tau in this line, the presence of detectable AT100 signal suggests that this form of tau was still localized within the axonal compartment. AT100 diverged between genotypes: While signal decreased over time in WT neurons, it progressively increased in S305N_C1 and C2 neurons (Fig. 3E).

In isoHet and isoHom S305N neurons, AT8 and CP13 signal was evident in both soma and axons. Levels increased in a dose-dependent manner relative to S305N allele copy number compared to their isoWT controls at DIV21 (Fig. 3, B, F, and G). At DIV28, both AT8 and CP13 levels were significantly elevated in isoHet and isoHom neurons. AT100 in the isoWT neurons mirrored the trend seen in WT neurons, showing a reduction over time. In isoHet neurons, the level of AT100 significantly increased at DIV28 (*P* = 0.0005), whereas in isoHom neurons, elevated AT100 signal was detected at both time points (Fig. 3H). Notably, unlike AT8 and CP13, AT100 did not exhibit a clear allele dose–dependent increase. Similarly, we also normalized AT8 and CP13 levels to TauC (fig. S4, D and F) where we can see that AT8/TauC levels reached significance only at DIV28 in the isoHom S305N. CP13/TauC also had a trend toward increased levels but did not reach significance because of variability.

Collectively, these results highlight distinct phosphorylation patterns and subcellular localization profiles of tau in neurons with elevated 4R tau, expressing the S305N mutation. Some phosphoepitopes, such as AT8, were restricted to the soma (e.g., in S305N_C1 and C2), others were distributed in both soma and axons (e.g., CP13), while AT100 was found exclusively in axons. Increased 4R tau in S305N mutant neurons therefore influenced isoform composition, the phosphorylation status, and the spatial distribution of tau proteoforms.

### Temporal analysis of total and pTau reveals early alterations in S305N mutant neurons

**Fig. 4.**
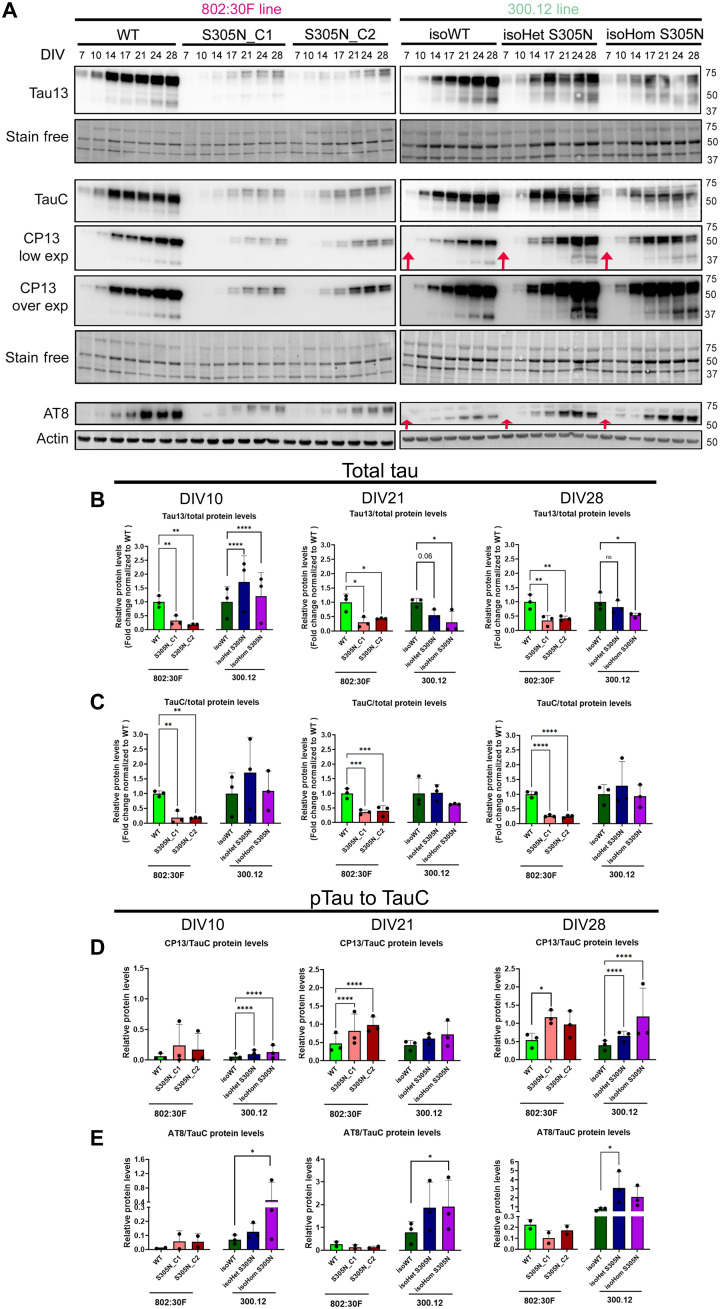
Temporal analysis of total and pTau in S305N i^3^N neurons. (**A**) Representative immunoblots showing total tau (Tau13: top; TauC: bottom) and pTau (CP13 and AT8, bottom) in all neuronal lines over time. Stain-free imaging or actin was used as a loading control, as indicated. Red arrows denote early phosphorylation events. (**B** and **C**) Quantification of total tau antibody levels: Tau13/total protein (B) and TauC/total protein (C) at DIV10, DIV21, and DIV28 across all lines. (**D** and **E**) Quantification of pTau antibody levels: CP13/TauC (D) and AT8/TauC (E) at DIV10, DIV21, and DIV28 across all neuronal lines. Statistical comparisons were made between mutant lines and their respective isoWT controls. Repeated measures one-way ANOVA with Dunnett’s post hoc test was used to compare S305N_C1 and C2 vs WT, and isoHet/isoHom S305N versus isoWT. Immunoblots for each line were performed separately. These tests were selected because of variability in immunoblot replicates. Data are presented as means ± SD; *P < 0.05, **P < 0.01, ***P < 0.001, ****P < 0.0001; *n* = 2 to 3 independent differentiations per presented neuronal line.

To determine whether the observed reduction in total tau levels in S305N_C1 and C2 neurons at DIV21 and DIV28 represents a developmental event or a progressive consequence of differentiation, we performed immunoblotting on lysates collected from DIV7 to DIV28. Blots were probed with antibodies against total tau (Tau13 and TauC) and pTau (CP13 and AT8; Fig. 4A). Across all time points, S305N_C1 and C2 neurons displayed consistently lower levels of total tau compared to WT controls (fig. S5, A and B). Tau protein expression in both WT and S305N_C1 and C2 neurons increased over time, mirroring MAPT mRNA expression patterns. As previous studies focused on DIV21 and DIV28, we specifically compared tau expression at these time points, as well as at DIV10, the earliest time point where tau was detected, with all antibodies used (Fig. 4, B, and C). In the isoHet S305N neurons, Tau13, was significantly increased at DIV10 (Fig. 4B, left graphs), whereas at DIV21 and DIV28, Tau13 levels presented a trend toward reduction compared to isoWT (*P* = 0.06; Fig. 4B, middle and right). In the isoHom S305N neurons, an ∼40% reduction in Tau13 was observed at DIV21 and DIV28, while TauC showed a trend toward reduced levels (∼20% reduction; Fig. 4, B and C). Notably, Tau13 and TauC changes mirrored immunocytochemistry results. A trend toward reduced total tau levels in the same lines was also observed in a previous study when using the direct differentiation approach to generate cortical neurons (*30*). In general, the reduction in total tau levels was seen in neurons expressing more than 50% 4R tau.

pTau levels showed a similar pattern to tau in the S305N_C1 and C2 neurons over time (fig. S5, C to F). CP13 expression was significantly reduced in S305N_C1 and C2 neurons when normalized to total protein at DIV21 and DIV28 (fig. S5, G to I). AT8 levels to total protein were reduced at DIV10; however, at DIV21 and DIV28, AT8 levels seem to increase and reach almost WT levels (fig. S5, G to I). When normalized to TauC, the reduction in CP13 was attenuated and a trend toward increased levels was observed at DIV10, which became statistically significant at DIV21 for both clones, and at DIV28 only for S305N_C1 compared to WT neurons (Fig. 4D). The ratio of AT8/TauC presented a small increase at earlier time points (DIV10) but declined thereafter (Fig. 4E). On the basis of immunocytochemistry, AT8/TauC levels were increased, possibly because of differences in detecting phosphorylated epitopes between techniques that can affect accessibility. Elevated CP13 levels normalized to total tau indicate tau hyperphosphorylation in S305N_C1 and C2 neurons.

Consistent with staining data, both CP13 and AT8 levels were elevated in isoHet and isoHom neurons over time (fig. S5, C to F). When normalized to total protein, CP13 expression increased in the isoHet S305N relative to isoWT controls at DIV10 and DIV28 only (fig. S5, G to I; graphs on the left). In the isoHom S305N, CP13 levels were also increased over time but did not reach significance because of variability, only reaching significance at DIV28 (fig. S5, G to I; graphs on the left). AT8 levels followed a similar pattern but did not reach significance at any time point because of variability (fig. S5, G to I; graphs on the right). Both CP13 and AT8 were also normalized to total tau. CP13/TauC was statistically significantly increased in isoHom and isoHet S305N neurons at DIV10 and DIV28 and presented a trend toward increased expression at DIV21 (Fig. 4D). Similarly, when AT8 levels were normalized to TauC, a significant increase in AT8 levels was observed in the isoHom S305N compared to isoWT neurons at DIV10 and DIV21 (Fig. 4E). AT8/TauC levels were increased in the isoHet S305N, but that change was only significant at DIV28 (Fig. 4E). A 4R MAPT–dependent increase in AT8/TauC levels was also noted at DIV10 and for CP13/TauC at DIV28 (Fig. 4E). The increase in CP13/TauC and AT8/TauC indicated that tau is hyperphosphorylated in the isoHet and isoHom S305N neurons. Notably, both AT8 and CP13 signals were detectable as early as DIV7 in both neuronal lines, indicating early tau phosphorylation (Fig. 4A red arrows, and fig. S5J).

These findings confirm that S305N_C1 and C2 neurons consistently express only 4R tau during differentiation. Although isoHet and isoHom neurons also showed reduced total tau after DIV21, both lines exhibited substantial pTau accumulation early in development. This early, and 4R MAPT–dependent phosphorylation of tau, particularly in the context of elevated 4R tau, may underlie the pathophysiological changes associated with mutations that increase exon 10 inclusion, as seen in postmortem human tissue (*26*, *28*, *29*, *41*, *42*).

### S305N mutant neurons develop endogenous tau oligomers and seeds

**Fig. 5.**
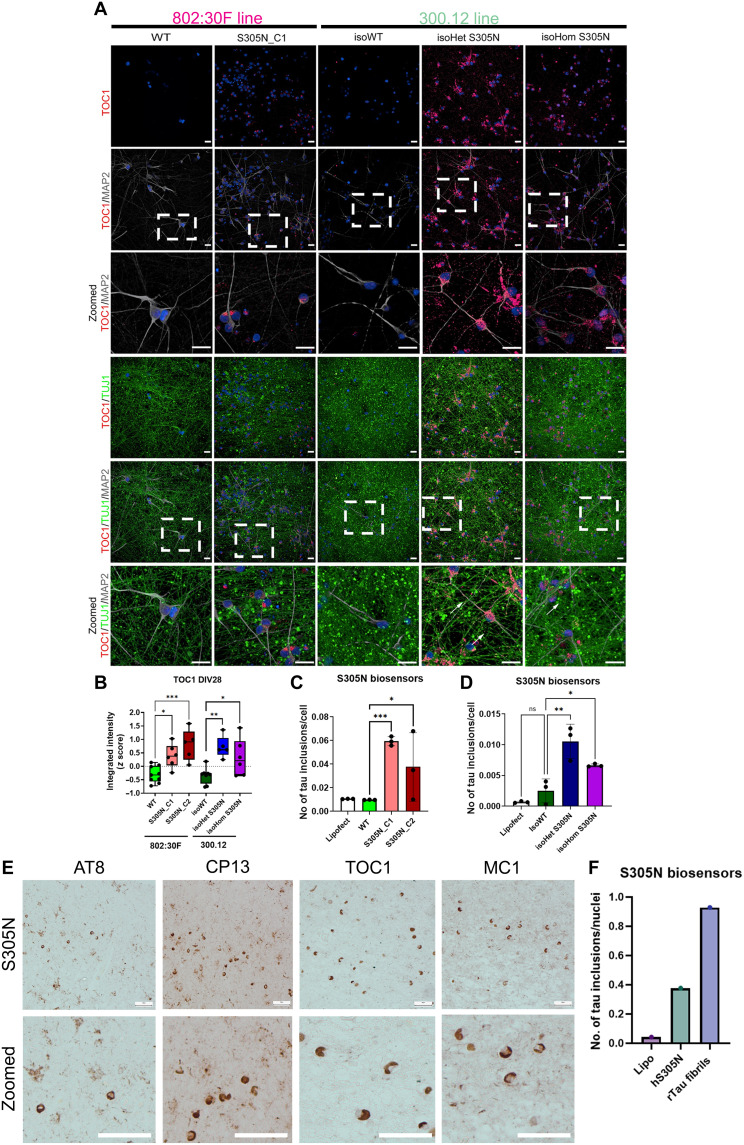
S305N i^3^N neurons form endogenous seed-competent tau. (**A**) Representative images of DIV28 i^3^N neurons colabeled with TOC1 and either MAP2 (top and zoomed-in) or TUJ1 (bottom and zoomed-in) across all neuronal lines. (**B**) Quantification of TOC1 labeling in DIV28 i3N neurons in the MAP2-positive area. Statistical analysis was performed using one-way ANOVA followed by Tukey post hoc test to compare mutant lines with their respective isogenic controls (mean ± SD, *n* = 4 to 5 independent differentiations per presented neuronal line). (**C** and **D**) Quantification of seeding activity at DIV28 in S305N biosensor line (one-way ANOVA followed by Tukey post hoc test; mean ± SD, *n* = 3 independent differentiations per presented neuronal line). (**E**) Representative images of human brain tissue from patient carrying the S305N mutation, stained for AT8, CP13, TOC1, and MC1. (**F**) Quantification of seeding activity from 3 μg of frozen human S305N brain tissue using the S305N biosensor assay. Statistical significance: *P < 0.05, **P < 0.01, ***P < 0.001.

To evaluate the aggregation potential of tau in S305N mutant neurons, we performed immunocytochemistry using the tau oligomer–recognizing antibody TOC1 developed by L. Binder’s laboratory (*43*), on methanol-fixed cells at DIV21 and DIV28. Neurons were colabeled with MAP2 and TUJ1 to assess subcellular localization. At DIV28, TOC1 signal was undetectable in WT and isoWT neurons; in contrast, neurons harboring the S305N mutation exhibited markedly increased TOC1 labeling (Fig. 5A and fig. S6C for S305N_C2). In S305N_C1 and C2 neurons, TOC1 signal was predominantly perinuclear and strongly colocalized with MAP2 (see enlarged MAP2/TOC1 panels). Notably, there was minimal colocalization with TUJ1 or tubulin blebs in these neurons. On the basis of that observation, only TOC1 expression was quantified in the MAP2-positive area. Both isoHet and isoHom S305N neurons also displayed elevated TOC1 labeling at DIV28, with isoHet neurons appearing to exhibit slightly higher levels. In these cells, TOC1 occupied a significant portion of the MAP2-positive area, with occasional puncta extending into TUJ1-positive regions. In some cases, these puncta colocalized with tubulin blebs (Fig. 5A, TUJ1/MAP2/TOC1 panels, arrows). Quantitative analysis confirmed a significant increase in TOC1 signal in all S305N-mutant neurons compared to their isoWT controls at DIV28 (Fig. 5B). To confirm that TOC1-positive puncta reflected accumulated tau, we performed costaining with Tau13. Colocalization between TOC1 and Tau13 was observed in DIV28 neurons, supporting the specificity of TOC1 for tau species (fig. S6C). At DIV21, TOC1 signal was also detectable and largely restricted to MAP2-positive somatodendritic compartments (fig. S6A). Colocalization of TOC1 with tubulin blebs was again observed in isoHom neurons (fig. S6B). A statistically significant increase in TOC1 signal was found in S305N_C1 and C2 and isoHet neurons at this earlier time point (fig. S7A). We also confirmed that the observed phenotype is not driven solely by a few TOC1-positive i^3^N neurons. To support this, we calculated the percentage of neurons with positive TOC1 staining within the MAP2-defined area and found that at both time points, all mutant lines exhibited more than 60% TOC1-positive staining (fig. S7B).

To assess whether S305N neurons developed tau seeds, we performed seeding assays using the P301S biosensors (*44*) and a modified imaging seeding assay [S305N biosensors (*31*)] using a tau biosensor cell line expressing either the repeat domain of tau with the P301S mutation fused to cyan fluorescent protein (CFP)/yellow fluorescent protein (YFP) or the S305N mutation fused to YFP. Eighteen micrograms of tris-buffered saline (TBS)–soluble cell extracts from DIV28 S305N_C1 and C2, isoHet, and isoHom neurons triggered a significant increase in tau inclusion formation compared to their respective isogenic WT controls (six and fourfold in S305N_C1 and C2, respectively, and four and threefold in the isoHet and isoHom S305N compared to respective isogenic WT controls; Fig. 5, C and D, and fig. S7C) in the S305N biosensor. With the P301S biosensor, we were also able to detect increased fluorescence resonance energy transfer (FRET) signal in all lines compared to WT; however, that change was only significant in the S305N_C1 line, probably because of variation between inductions (fig. S7, D and E). In contrast, extracts from DIV21 neurons did not induce seeding activity (fig. S7, F and G), suggesting that seed-competent tau develops later in neuronal maturation. Collectively, these results indicate that S305N mutant neurons develop robust TOC1-positive assemblies and early species of seed-competent tau species capable of propagating aggregation in a cellular biosensor model. Although S305N lines had lower tau levels compared to their respective control lines, they were able to form seeds independent of the amount of tau in the lysates.

Postmortem human frontal cortex brain tissue from an S305N mutation carrier demonstrated tau pathology consistent with our findings in i^3^N neurons. Neurons in the human S305N case were positive for AT8, CP13, TOC1, and MC1 (Fig. 5E). Ring-shaped tau aggregates surrounding neuronal nuclei were visualized using TOC1 and MC1 antibodies. These observations are consistent with previously reported pathological features identified in human S305N FTDP-MAPT tissue (*26*, *28*, *29*, *31*, *41*, *42*). Three micrograms of TBS-soluble extract from S305N human brain tissue was able to template the aggregation of tau in the S305N biosensor line (Fig. 5F and fig. S7H) and the P301S line (fig. S7I). Overall, our S305N i^3^N neurons recapitulate key aspects of human pathology except for MC1 immunoreactivity. Given the relatively short culture times, these cells may therefore reflect the earliest tau-related pathological changes observed in humans.

### Live imaging reveals progressive accumulation of tau assemblies in S305N neurons

**Fig. 6.**
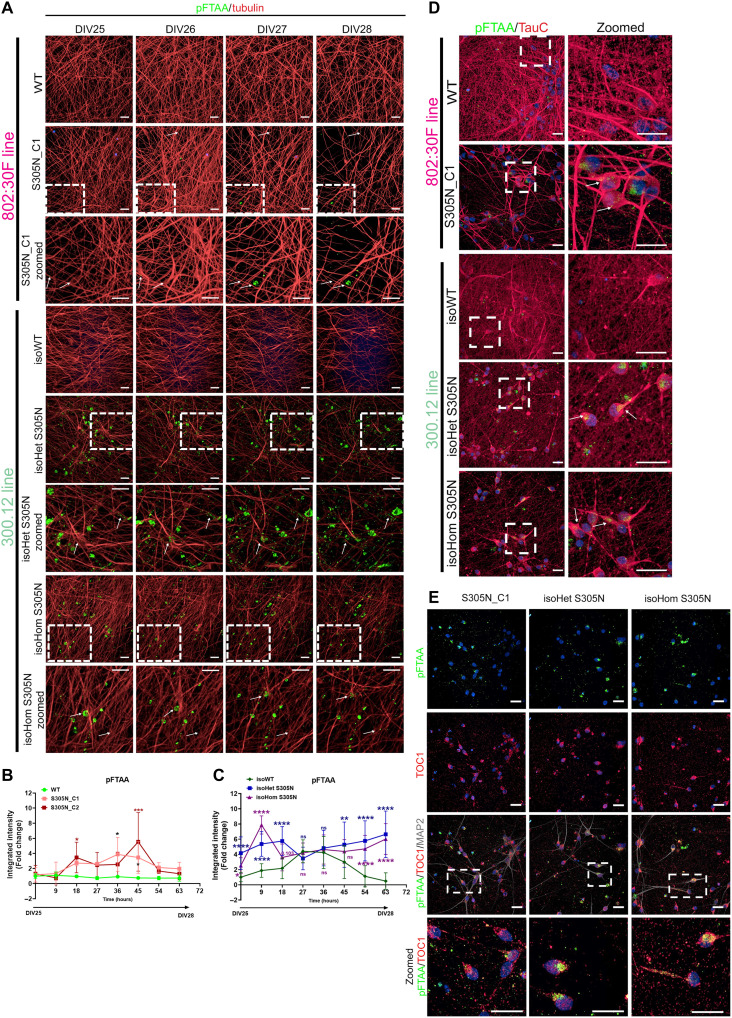
pFTAA-positive inclusions in S305N i^3^N neurons. (**A**) Representative live-cell images of i^3^N neurons labeled with pFTAA and tubulin over time. Dyes were added at DIV25, and images were acquired every 9 hours. (**B**) Quantification of pFTAA signal within tubulin-positive regions (surrounding the nuclei) over time in WT and S305N_C1 and C2 neurons (two-way ANOVA followed by Tukey post hoc test; mean ± SD, *n* = 3 independent differentiations per presented neuronal line). (**C**) Quantification of pFTAA signal within tubulin-positive regions over time in isoWT, isoHet, and isoHom S305N neurons (two-way ANOVA followed by Tukey post hoc test; mean ± SD, *n* = 3 independent differentiations per presented neuronal line). (**D**) Representative images of DIV28 neurons fixed with methanol and co-labeled with pFTAA and TauC. (**E**) Representative images showing colocalization of pFTAA with TOC1 and MAP2 in DIV28 neurons fixed with methanol. Statistical significance: *P < 0.05; **P < 0.01; ***P < 0.001; ****P < 0.0001.

Seeding assays indicated that neurons carrying the S305N mutation develop seed-competent tau by DIV28 but not at DIV21. Given that TOC1-positive signal was detected at both time points, we next sought to monitor the longitudinal formation and progression of tau assemblies in live neuronal cultures. To accomplish this, we used the conformation-sensitive fluorescent dye pentameric formyl thiophene acetic acid (pFTAA), which selectively binds to β sheet–rich protein aggregates, including both early tau oligomers and mature fibrils (*45*, *46*). Previous studies have shown that pFTAA is nontoxic and remains stable in primary neuronal cultures for extended periods with routine media replacement (*46*). pFTAA and a far-red live tubulin dye were applied to neurons at DIV25, and the accumulation of tau assemblies was imaged over a 72-hour period (Fig. 6A). In S305N_C1 and C2 neurons, pFTAA-positive puncta were first observed within axonal processes, identified by tubulin staining. Over time, larger aggregates formed, predominantly in preaxonal segments or in proximity to the nucleus (arrows in enlarged panel, Fig. 6A and fig. S8A for S305N_C2). Quantification revealed that pFTAA-positive puncta began to emerge approximately 18 hours after dye application and progressively increased over time in the S305N_C1 and C2 neurons (Fig. 6B). At later time points, the pFTAA signal is reduced (although it remains above WT levels), which likely reflects the reduction in tubulin staining over time, limiting our ability to quantify pFTAA signal outside of tubulin-positive regions. Similarly, isoHet and isoHom S305N neurons also showed pFTAA accumulation, but as early as 30 min after incubation (DIV25), indicating a more rapid onset of aggregate formation or that aggregates are already present at the starting timepoint (Fig. 6A). Some pFTAA-positive structures appeared in regions formerly occupied by viable neurons, which could indicate that the structures were associated with neuronal death, while others extended from preexisting pFTAA accumulations. These puncta were consistently observed in tubulin-rich regions and along axon-like projections (Fig. 6A, arrows). Quantitative analysis confirmed a steady increase in pFTAA signal in both isoHet and isoHom S305N neurons throughout the imaging period (Fig. 6C).

As pFTAA is not specific for tau, we confirmed that the pFTAA signal seen in the S305N cells represented tau assemblies by costaining methanol-fixed neurons with TauC (total tau) and TOC1 (oligomeric tau). In S305N-mutant lines, pFTAA and TauC immunolabeling co-occur in the same region of the cell, which is predominantly perinuclear (Fig. 6D and fig. S8A for S305N_C2). As both TOC1 and pFTAA can label dying neurons, we costained with MAP2 to assess neuronal viability. Most pFTAA- and TOC1-positive neurons also labeled positively for MAP2, suggesting that they were still viable and part of the neuronal cell population (Fig. 6E and fig. S8A for S305N_C2). Further, colocalization of pFTAA with Lysotracker Red in S305N_C1, S305N_C2, isoHet, and isoHom S305N neurons revealed that tau assemblies were accumulating in lysosomes at DIV28 rather than being degraded (fig. S8B). These results demonstrate that tau assemblies in S305N neurons develop progressively over time, with isoHet and isoHom neurons exhibiting earlier and more extensive aggregate formation than S305N_C1 and C2. The pFTAA-positive structures likely represent early oligomeric and potentially protofibrillar tau species that accumulate in tubulin-rich compartments and evade lysosomal degradation, contributing to tau pathogenesis in S305N mutant neurons.

### Cytoskeletal alterations in S305N neurons

**Fig. 7.**
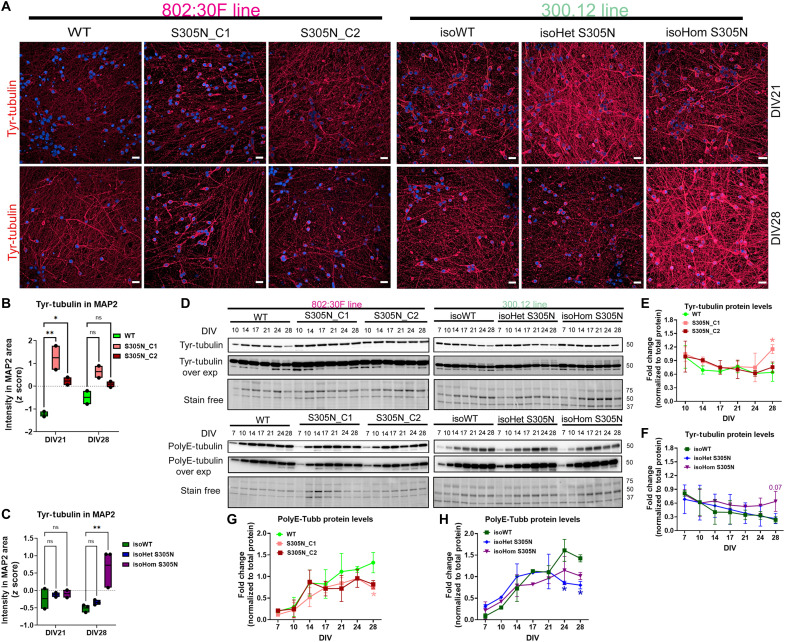
S305N i^3^N neurons demonstrate markers of cytoskeleton changes. (**A**) Representative immunostaining of Tyr-Tubulin in i^3^N neurons at DIV21 and DIV28 across all neuronal lines. (**B**) Quantification of Tyr-Tubulin signal within MAP2-positive regions in WT versus S305N_C1 and S305N_C2 neurons at DIV21 and DIV28. Statistical analysis was performed using two-way ANOVA followed by Tukey’s post hoc test (mean ± SD, *n* = 2 independent differentiations per presented neuronal line). (**C**) Quantification of Tyr-Tubulin signal within MAP2-positive regions in isoWT versus isoHet and isoHom S305N neurons at DIV21 and DIV28. Statistical analysis was performed using two-way ANOVA followed by Tukey’s post hoc test (mean ± SD, *n* = 2 to 3 independent differentiations per presented neuronal line). (**D**) Representative immunoblots showing Tyr-Tubulin (top) and polyglutamylated tubulin (PolyE Tubulin; bottom) expression over time in all neuronal lines. (**E**) Quantification of Tyr-Tubulin protein levels in WT versus S305N_C1 and C2 neurons over time, normalized to total protein. Statistical analysis: two-way ANOVA with Tukey’s post hoc test (mean ± SD, *n* = 2 independent differentiations per presented neuronal line). (**F**) Quantification of Tyr-Tubulin protein levels in isoWT versus isoHet and isoHom S305N neurons over time, normalized to total protein. Statistical analysis: two-way ANOVA with Tukey’s post hoc test (mean ± SD, *n* = 2 independent differentiations per presented neuronal line). (**G**) Quantification of PolyE Tubulin levels normalized to total protein over time in (G) WT versus S305N_C1 and C2 neurons and (**H**) in isoWT versus isoHet and isoHom S305N neurons. Statistical analysis: two-way ANOVA with Tukey’s post hoc test (mean ± SD, *n* = 2 independent differentiations per presented neuronal line). Statistical significance: *P < 0.05, **P < 0.01.

Given the known role of tau in microtubule dynamics, we assessed posttranslational modifications (PTMs) of tubulin using immunocytochemistry and immunoblotting. Tyrosinated α-tubulin (Tyr-Tubulin), a marker of dynamic and newly polymerized microtubules (*47*, *48*), was more prominently localized in the somatodendritic compartment of S305N-mutant neurons (Fig. 7A). Quantitative analysis revealed a significant increase in Tyr-Tubulin signal within MAP2-positive regions of S305N_C1 and C2 neurons at DIV21, whereas at DIV28 only S305N_C1 presented a close to significant change (*P* = 0.06), with isoHom neurons showing elevated levels only at DIV28 (Fig. 7, B and C). Longitudinal quantification of Tyr-Tubulin levels showed a progressive decline in WT and isoWT neurons, consistent with the transition toward stable axonal architecture during maturation. Our results indicate that S305N lines with a homozygous mutation had a trend toward elevated Tyr-Tubulin levels compared to their respective WT controls, which could indicate altered microtubule dynamics or delayed axonal stabilization (Fig. 7, D to F). We also investigated tubulin polyglutamylation (PolyE), a PTM that enhances microtubule rigidity and resistance to depolymerization (*49*). In WT and isoWT neurons, PolyE tubulin levels increased over time, consistent with the formation of stable microtubules. However, S305N_C1 and C2 neurons start presenting a decrease in PolyE tubulin from DIV17; this change was only significant at DIV28. The isoHet and isoHom S305N neurons had significantly lower levels of polyE tubulin from DIV24, and the change was only statistically significant in the isoHet line (Fig. 7, D to H). Although the cytoskeleton changes were only observed in the mutant lines, the precise relationship with tau type and distribution will require more extensive testing.

### A novel neuronal model that responds to tau modulators

**Fig. 8.**
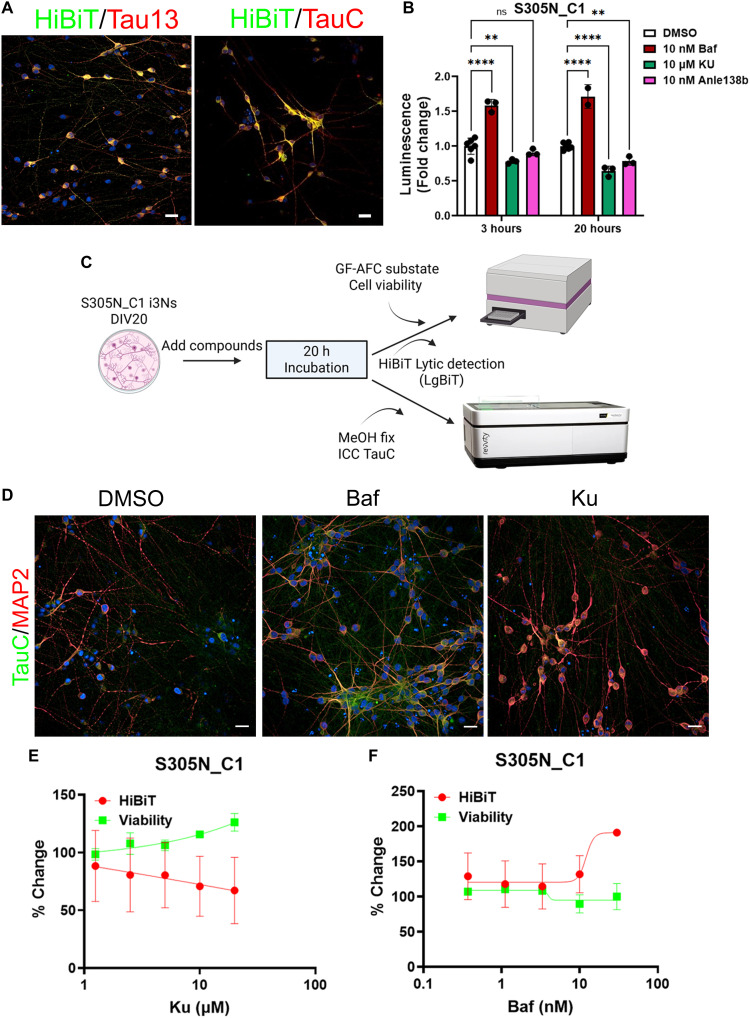
Optimization of a drug screen assay for tau modulators. (**A**) Representative images showing colocalization of the HiBiT tag with Tau13 (left) and TauC (right) in DIV21 neurons. (**B**) Luminescence changes following treatment with dimethyl sulfoxide (DMSO), 10 nM bafilomycin A1 (Baf), 10 μM KU-0063794 (Ku), or 10 nM Anle138b for either 3 or 20 hours (h) at DIV21. Statistical analysis was performed using two-way ANOVA followed by Tukey’s post hoc test (mean ± SD, *n* = 3 independent differentiations per presented neuronal line with three wells per condition per differentiation). (**C**) Schematic representation of the proposed screening platform for 4R tau modulators [Created in BioRender. Tsefou, E. (2026) https://BioRender.com/nh7i3lr]. (**D**) Representative images of methanol-fixed DIV21 neurons stained with TauC and MAP2 after 20-hour treatment with DMSO, 10 nM Baf, or 10 μM Ku. (**E** and **F**) Dose-response curves showing changes in luminescence (HiBiT signal) and cell viability in DIV21 S305N_C1 neurons treated with Ku (E) or Baf (F) for 20 hours. Data represents mean ± SD from two independent differentiations per presented neuronal line, with three wells per condition per experiment. Statistical significance: **P < 0.0, ****P < 0.0001. h, hours.

A major limitation in the field is the lack of a high-throughput screening assay to monitor the levels of endogenous tau in human iPSC-derived neurons. To address this, we inserted an 11–amino acid peptide (HiBiT) that allows sensitive luminescence-based detection of tagged proteins when complemented with LargeBiT (LgBiT) in a lytic luminescence assay. CRISPR-Cas9–mediated genome editing was used to insert the HiBiT tag at the C terminus of the MAPT gene in S305N_C1, which was chosen as it presented the strongest phenotype regarding seed-competency and TOC1/pFTAA accumulation. Successful integration and expression of HiBiT-tagged tau was confirmed by immunocytochemistry in methanol-fixed neurons using Tau13 and TauC antibodies, both of which colocalized with the HiBiT signal at DIV21 (Fig. 8A). To test the potentials benefits of the HiBiT tag, we treated neurons with a panel of compounds known to affect tau dynamics, including the autophagy inhibitor bafilomycin A1 (Baf), the mechanistic target of rapamycin (mTOR) inhibitor KU-0063794 (KU), and the small molecule Anle138b, reported to inhibit tau aggregation (*50*). After 3 hours of treatment, Baf led to a 50% increase in HiBiT signal, while KU led to a reduction. These effects were more pronounced after 20 hours, with Baf significantly increasing HiBiT signal by almost twofold and both KU and Anle138b showing more than 30% reduction (Fig. 8B) compared to dimethyl sulfoxide (DMSO)–treated cells. On this basis, we selected the 20-hour time point for subsequent screening. The HiBiT lytic assay only monitors the amount of HiBiT-tagged tau and not the abundance or cellular distribution of tau proteoforms, which are important aspects of tau pathogenesis. Characterization of S305N_C1 i^3^N neurons had revealed that tau relocated from axonal to somatodendritic compartments (Fig. 2B), a redistribution that is known to increase its propensity for aggregation due to reduced microtubule binding. Compounds that can prevent this or modulate the levels of tau overall may be therapeutically relevant. We therefore believe that a platform combining the HiBiT luminescence assay with high-content imaging for phenotypic screening will be beneficial for the field. This setup will enable parallel assessment of compound effects on tau levels (via the lytic HiBiT assay), tau localization (via TauC immunostaining), and cell viability (Fig. 8C). As a proof of concept for this platform, we treated S305N_C1 neurons with Baf or KU for 20 hours. TauC staining confirmed increased tau accumulation with Baf, including a potential increase in axonal tau, while KU treatment resulted in reduced tau staining, consistent with enhanced clearance (Fig. 8D). Dose-response experiments with KU and Baf showed concentration-dependent modulation of the HiBiT signal (Fig. 8, E and F), demonstrating the assay’s sensitivity and dynamic range. Notably, neither compound adversely affected cell viability under these conditions; in fact, high concentrations of KU slightly increased viability (∼25%), suggesting a potential protective effect. Collectively, these results demonstrate that the HiBiT-tagged S305N_C1 neuronal model has the potential to be a robust model for quantifying changes in tau levels as well as cellular distribution, for the screening of tau-modulating compounds in a high-throughput manner.

## DISCUSSION

The human neuron cell lines we describe here represent a significant advance for the neurodegeneration field as they recapitulate several of the key pathogenic events observed in human diseases associated with tauopathy. One of the major problems in the field has been that most iPSC-derived human neurons, even those expressing FTD-causing mutations, model the fetal state and do not accumulate appreciable levels of 4R tau, and they do not form endogenous seed-competent tau assemblies. Human neuronal lines that do accumulate 4R tau require multiple mutations or genetic modification to increase 4R tau expression, or extended culturing time. Tau aggregates usually have to be induced by incubation with exogenous tau seeds or by removing splice elements and inserting combinations of mutations that do not co-occur in human (*22*). All of these approaches limit physiological relevance (*17*–*22*). Our data, from two independent human donor lines, demonstrate that expressing the S305N mutation leads to rapid, elevated 4R tau accumulation, which is associated with altered tau phosphorylation, distribution, aggregation, and downstream cellular pathways critical for neuronal homeostasis and microtubule stability (see Summary, table S1), recapitulating what is observed in a wide range of human tauopathies.

Clinically, S305N-linked FTDP-MAPT is associated with 4R tau pathology, ring-shaped perinuclear aggregated tau, and neurofibrillary tangles (*33*, *41*, *42*). Our neuronal lines recapitulate much of this phenotype (including seed-competent tau), and we believe that they represent an early stage of this pathogenic process, albeit one that arises in a developmental context where 4R tau is not normally expressed. Whether the effects observed in S305N mutant lines mirror the pathological events underlying other MAPT mutations, sporadic primary tauopathies, secondary tauopathies, and inflammation-driven tauopathies (*51*), or other neurodegenerative disorders is difficult to confirm. However, this may be irrelevant if heterogeneous etiologies converge on the common pathological pathways of tau hyperphosphorylation and aggregation.

A consistent finding across both the CRISPR-edited and patient-derived mutant lines was the reduction in total tau levels, which has important implications for interpreting phosphorylation and seeding data. The reduction in total tau may itself represent a mutation-associated phenotype, potentially reflecting altered tau stability, increased degradation, or transcriptional regulation. To account for this, phosphorylation measurements were interpreted relative to total tau levels allowing assessment of modification state independent of overall protein abundance. The levels of pTau were significantly altered in the mutant neuronal lines, particularly in S305N neurons with the homozygous mutation. This was demonstrated by increased CP13 and/or AT8 immunoreactivity, regardless of overall reduced total tau levels. However, we observed that despite the overall relatively lower levels of 4R tau, isoHet S305N neurons presented a trend toward higher TOC1, pFTAA, and seed competency compared to isoHom S305N, suggesting that the relationship between 4R levels, phosphorylation status, and aggregate formation is not direct. Increased phosphorylation in the same isoHom line has been reported; however, the authors did not measure changes in seeding activity or accumulation of TOC1/pFTAA (*30*). We observed interesting differences in the distribution of several pTau epitopes, with AT100 only being detected in the axons and not in the somatodendritic compartment. The AT100 (pThr^212^/pSer^214^) phosphorylation sites are thought to be critical for modulating the ability of tau to bind and maintain microtubule stability (*52*–*56*). Tau hyperphosphorylation at the AT100 epitope may promote or indicate tau detachment from microtubules, increasing its propensity to form small soluble oligomers or insoluble aggregates, which could be toxic to neurons (*52*–*56*). AT100 was increased at DIV28 in all neuronal lines harboring the S305N mutation, independent of the amount of total tau, and at a time point when pFTAA positive signal was present and neurons had formed endogenous, seed-competent tau. The presence of increased AT100 immunoreactive tau in axonal compartments might indicate that tau oligomer formation is initiated there and that tau seeds are then transported to the soma, where we see the highest accumulation of TOC1 immunoreactive tau and pFTAA signal. AT8 is the only pTau antibody located in the soma, and it is similar to TOC1/pFTAA distribution.

Notably, longitudinal imaging revealed that tau assemblies accumulate in axons before the soma, suggesting that the axons are the initial point of vulnerability. The appearance of TOC1-positive oligomeric tau, particularly in the perinuclear region and in MAP2-positive compartments, indicates the presence of aggregation-prone tau species in these compartments. Similar structures are present in neurons in the cortex of human S305N brain tissue. The high density of i^3^N cultures limited our ability to consistently resolve pFTAA-positive puncta along axons; however, we observed discrete puncta predominantly near nuclei and within preaxonal segments. At DIV28, pFTAA signal was largely confined to MAP2-positive regions and colabels with TauC, confirming that pFTAA was labeling abnormal conformers of tau. The colocalization of pFTAA-positive structures with lysosomes suggests that these assemblies are resistant to degradation, consistent with the behavior of oligomeric tau (*57*, *58*). However, the absence of tau labeling by MC1, a conformation-specific antibody recognizing tau in neurofibrillary tangles, suggests that despite the accumulation of oligomeric, seed-competent tau in our models, the formation of more mature tangle-like pathology may require longer culture times, the presence of mixed neural and glial populations or additional stressors that more closely mimic the in vivo disease environment. The presence of tau seeds in DIV28 neuronal lysates, as shown by the biosensor assay, suggests that S305N neurons not only accumulate misfolded tau but also generate seed-competent species capable of propagating pathology. The absence of seeding at DIV21, despite a detectable TOC1 signal, implies a temporal progression from early oligomeric intermediates to seed-competent assemblies. However, overall, the seeding signal was low, possibly because of quite low levels of tau in the neurons, which makes it difficult to identify small changes at DIV21 compared to DIV28. Of note, our longitudinal studies support a model where tau misfolding and phosphorylation precede the formation of seed-competent tau species.

Biochemical and morphological data, which include the presence of tubulin blebbing, reduced MAP2 levels, and reduced VGLUT1 expression, highlight the functional consequences of tau dysregulation in the i^3^N neurons. PTMs of tubulin provide additional insight into the cellular impact. Elevated tyrosinated tubulin and reduced polyglutamylation in the neurons with more than 75% 4R tau suggest a shift toward a more dynamic, less stable microtubule network. These changes are consistent with a failure to mature structurally stable axons. In human AD brain tissue, reductions in acetylated (*59*) and polyglutamylated (*49*) α-tubulin has been reported, suggesting that tau dysregulation affects microtubule structure both in vitro and in vivo (*60*).

One of the key current limitations for drug discovery targeting tauopathies is the absence of a high-throughput assay to quantify 4R tau dynamics and functional outcomes in living, human neurons. To address this, we developed a luminescence-based assay by knocking in a HiBiT tag at the endogenous *MAPT* C terminus in the S305N_C1 iPSC line. This system enables rapid, sensitive detection of endogenous tau levels and offers the added benefit of compatibility with drug screening pipelines. Using the lytic assay, we tested the effects of known tau-modulating compounds. Bafilomycin A1 treatment increased HiBiT signal, reflecting tau accumulation, whereas KU-0063794 and Anle138b reduced tau levels in a time- and concentration-dependent manner. This system will also allow phenotypic readouts through high-content imaging using the HiBiT tag system, enabling the identification of compounds that not only alter overall tau (C terminus) levels, but also compounds that affect the subcellular localization of different tau proteoforms, a key pathological feature of human tauopathies that is represented in our cell model. Notably, these lines are the first single coding mutation i^3^N neuronal line to report formation of endogenous tau assemblies, an important target of therapeutics, which can be monitored longitudinally in these lines. On the basis of our results, expansion of the toolbelt of S305N mutant lines (including the development of a KOLF line for cross-lab comparison), is now justified.

In conclusion, our study provides insight into how the accumulation of 4R human tau caused by the S305N mutation disrupts the normal biological function of tau, leading to pathological changes including altered tau, increased phosphorylation and aggregation, impaired microtubule stability, and dysregulation of key neuronal pathways. The reasonable timeframe and progressive nature of the tau pathology observed in these neuronal cell lines will make them a valuable tool for assessing the sequence of pathological events during the initial stages and early progression of 4R tauopathies. In addition, being able to assess the order of events and the spatiotemporal relationship between cellular location and tau proteoform distribution, in the absence of overexpression artifacts or prolonged culturing, is a significant strength of this model. Our findings not only underscore the multifaceted role of tau in neuronal development and maintenance but also highlight potential therapeutic targets for the earliest stages of tau pathogenesis, and they provide proof-of-concept data for an assay platform to screen them.

### Limitations of the study

One limitation of our model is that we used the piggyBac system to introduce hNGN2 into the iPSC lines, which typically results in random integration into the genome. No karyotype abnormalities were observed, but there is still the possibility that hNGN2 could have disrupted some genes in these cells. Two observations could affect the interpretation of the study. First, the S305N_C1 and C2 neurons express 4R tau from a relatively early stage of neuronal differentiation, which could affect developmentally sensitive events. It is not known if 4R tau predominates during early life in humans expressing the S305N mutation. Second, there was a marked reduction in total tau protein levels in mutant lines compared to their isogenic WT controls. We do not believe that this is an artifact related to the donor line as the effect was seen in two clones from one of the lines, and in two different donor lines, with the isoHom S305N presenting a similar phenotype to S305N_1 and C2 neurons, albeit at a slightly later time point (after DIV21). As this corresponds to the time when 4R tau levels increase by more than 50%, the shift toward excessive 4R tau is likely implicated in disrupted protein turnover and axonal destabilization. A previous study has shown that 4R tau isoforms have a quicker turnover rate than 3R tau in iPSC-derived cells (*61*), which could result in lower overall tau levels in the mutant lines compared to the predominantly 3R-expressing WT line. Our observations are consistent with those published previously for the 300.12 lines (*30*).

## MATERIALS AND METHODS

### Generation of iPSC line with an S305N/IVS10 + 3 mutation via CRISPR-Cas9

iPSCs with mutations S305N and IVS10 + 3 were generated by Synthego using one of their in-house iPSC lines (802:30F). Briefly, a single guide (GUACUCACACUGCCGCCUCC) was used to introduce both mutations by using their in-house CRISP-Cas9 editing protocol. Sanger sequencing was used to identify clones with homozygote double mutation. Consequently, one of the clones (Clone B11) carrying the double homozygote mutation was selected to knock in the HiBiT tag in the C terminus of *MAPT*. Two clones were provided (D4 and G8) by Synthego that contained the double mutant and the HiBiT tag (S305N_C1 and C2) as well as the mock-transfected WT cells. The top three off-target sites for both single guide and HiBiT tag were assessed via PCR and sequencing. No off-target sites were identified.

### Culture of iPSCs

iPSCs on the 802:30F background were maintained in Geltrex (Thermo Fisher Scientific; A1413301) coated plates using mTeSR Plus (Stem Cell Technologies; 100-0276) and passaged using 0.5 mM EDTA (Thermo Fisher Scientific; 15575-020). Patient-derived iPSCs on the 300.12 background were obtained from NCRAD. These cells were maintained in Matrigel (Corning, 354277) using mTeSR1 (Stem Cell Technologies; 85850) and passaged using ReLeSR (Stem Cell Technologies; 100-0483). All iPSC lines for this work were regularly tested for mycoplasma.

### Stable integration of piggyBac plasmids into iPSCs

To generate stably expressing hNGN2-expressing iPSC, we integrated a blue fluorescent protein (BFP)–containing doxycycline-induced hNGN2 cassette using the piggyBac system. iPSCs were washed with phosphate-buffered saline (PBS), dissociated with Accutase (Gibco; A1110501), and plated into coated plates (Geltrex or Matrigel) at 1 × 10^6^ cells per well of a six-well plate in mTeSR Plus or mTeSR1 containing 10 μM Y-27632 (Tocris; 1254/10). Once cells were attached, they were washed with PBS before being transfected with constructs containing the donor doxycycline-induced hNGN2 and BFP fluorescent reporter flanked by transposon terminal repeats and the piggyBac transposase. For the transfection, 3 μg of total plasmid DNA was used (2:1 donor to transposase ratio), with Lipofectamine Stem (Thermo Fisher Scientific; STEM00008) as per the manufacturer’s instructions. Forty-eight hours posttransfection, the media were changed and supplemented with puromycin (1 μg/ml) for 3 days to select for cells that had successfully integrated the hNGN2. To obtain a pure population of cells with high levels of BFP expression due to cassette insertion, the cells were subjected to fluorescence-activated cell sorting using BFP fluorescence. To ensure genomic stability, iPSCs were karyotyped using low-coverage sequencing (UCL Genomics) with 50-kbp resolution. All lines were also checked routinely for abnormalities using the hiPSC Genetic Analysis Kit (Stem Cell Technologies, 07550).

### iPSC differentiation into cortical i^3^N neurons

Induction of iPSC into cortical neurons was initiated by dissociating cells with Accutase and plating 1 × 10^6^ iPSC onto Geltrex (Synthego 802:30F lines) or Matrigel (NCRAD 300.12 lines) coated six-well plates using Dulbecco’s modified Eagle’s medium (DMEM)/F-12 with Glutamax/Hepes (Thermo Fisher Scientific, 11330032) containing doxycycline (Sigma-Aldrich, 2 μg/ml; D9891), 1× N2 Supplement (Thermo Fisher Scientific, 17502048), and 1× nonessential amino acids (Thermo Fisher Scientific, 11140050) and 10 μM Y-27632. The media were replaced daily for the next 2 days with media without Y-27632. At day 3, induced cells were dissociated with Accutase and replated into plates double-coated with poly-l-ornithine (0.1 mg/ml; Sigma-Aldrich, P3655; 24 hours at 37°C) and laminin (30 μg/ml; R&D Systems, 3446-005-01; 24 to 72 hours at 37°C). Cells were plated into 6-well plates at 0.6 × 10^6^ cells per well, 24-well plates at 0.125 × 10^6^ cells per well, into 96-well Phenoplates (Revvity; 6055300) or white plates for Luminescence assays (Greiner, 655083) at 30,000 cells per well. Neuronal maturation media consisted of Neurobasal (Thermo Fisher Scientific, 21103049) media containing 1× B27 (Thermo Fisher Scientific, 17504044), brain-derived neurotrophic factor (BDNF, 10 ng/ml; PeproTech, 450-02), neurotrophin-3 (NT-3, 10 ng/ml, PeproTech, 450-03), laminin (6-12 μg/ml), doxycycline (2 μg/ml), and 1× CultureOne (Thermo Fisher Scientific, A3320201). Once a week, one-half of the media was replaced with fresh media until the cells were collected.

### Immunocytochemistry, imaging, and analysis

Human iPSCs were fixed with 4% methanol-free paraformaldehyde (Thermo Fisher Scientific; 28908) diluted in PBS for 15 min at room temperature and washed three times in Dulbecco’s phosphate-buffered saline (DPBS) (Thermo Fisher Scientific; 14190-094). Most of the i^3^N neurons were fixed with 100% methanol for 15 min at −20^o^ C. Cells were washed twice with ice-cold DPBS. Cells were blocked/permeabilized with Intercept Blocking Buffer (LICOR; 927-60003) containing 0.1% Triton X-100 (Sigma-Aldrich; 93443) for 1 hour at room temperature. Primary antibodies diluted in blocking buffer were added to the cells and incubated overnight at 4°C and then washed twice with DPBS. The following primary antibodies were used: chicken anti-MAP2 (1:2000, Novus; NB300-213), mouse anti-TUJ1(1:2000, Biolegend; 801202), chicken anti-NeuN (1:500, Millipore; ABN9), mouse anti-Tau13 (N terminus tau, 1:1000, Santa Cruz Biotechnologies; sc-21796), rabbit anti-TauC (C terminus tau, 1:5000, Daco; A0024), mouse anti-AT8 (1:500, Invitrogen; MN1020), mouse anti-CP13 (1:500, Peter Davies), mouse anti-TOC1 (1:1000, Nicholas M. Kanaan), mouse anti-AT100 (1:1000, Invitrogen; NM1060), rabbit anti-VGLUT1 (1:500, Millipore; MAB5502), mouse anti-HiBiT (1:500, Promega, N7200), rat anti-Tubulin (Tyr-Tubulin) (1:2000, Sigma-Aldrich; MAB1864), mouse anti–TRA-1-60 (1:2000, Abcam; Ab16289), mouse anti-SOX2 (1:1000, Santa Cruz Biotechnologies; sc-365823), mouse anti–TRA-1-81 (1:2000, Abcam; Ab16289), and rabbit anti-NANOG (1:2000, Cell Signaling Technologies; 4903S). Secondary antibodies diluted in blocking buffer, including goat anti-mouse, anti-rabbit or anti-chicken conjugated with Alexa Fluor 488, Alexa Fluor 568, or Alexa Fluor 647 (1:1000) as well as Hoechst (1:1000, Thermo Fisher Scientific; 33342) were added into the cells and incubated for 1 hour at room temperature. Cells were washed twice with DPBS. More than three independent inductions were performed for each line, and for each line, three to four technical replicates were included/induction.

Images were acquired with the high-content microscope Opera Phenix Plus (Revvity) by using the 40× water immersion objective with 12 fields of view per well and z-stack of six planes (0.5 μm per plane). Acquisition settings (exposure times and laser power) were adjusted to be above the camera background and kept below saturation levels. Imaging settings were kept the same between biological replicates.

Acquired images were analyzed in Harmony 5.1. Briefly, a stack of each image was maximum projected and basic flatfield correction was applied. Cells were segmented by identifying the nuclei of live cells only (“find nuclei building block”). “Find image region” was used to identify tau or tubulin in the imaged field. For antibodies where staining was localized in the soma, such as TOC1 or AT8, MAP2 was used to identify cytoplasm, and, consequently, “find image region with absolute thresholding” was used to identify the TOC1-positive area. In some cases, TOC1 labeled dead cells, and a filter was applied to remove these areas during the analysis. Both intensity and area of the studied protein were calculated, and the results were normalized to the number of cells. Integrated intensity was calculated by multiplying the area by the mean intensity. Because of variation in the absolute values of the integrated intensity between biological replicates, *z* scoring was used instead. For each biological experiment, the *z* score was calculated with the formula $Zi=\frac{Xi-\mu }{\sigma }$, where *X*_i_ is the value of the individual technical replicate, μ is the mean of all conditions for the specific biological experiment, and σ is the SD for all conditions for the specific biological experiment. Average *Z* scores from different biological replicates were plotted in GraphPad Prism.

### pFTAA live imaging of i^3^N neurons and imaging analysis

To monitor pFTAA staining in i^3^N neurons, cells were grown until DIV25. Old media were aspirated, and the cells were washed with fresh Neurobasal media without Phenol Red (Thermo Fisher Scientific; 12348017) containing 1× B27 (Thermo Fisher Scientific, 17504044), BDNF (10 ng/ml; PeproTech, 450-02), NT-3 (10 ng/ml; PeproTech, 450-03), laminin (6 to 12 μg/ml), doxycycline (2 μg/ml), and 1× CultureOne (Thermo Fisher Scientific, A3320201) to remove dead cell from the culture. Fresh neuronal media containing 1.5 μM pFTAA (Sigma-Aldrich, SCT066) and 1:10,000 Tubulin Tracker Deep Red (Thermo Fisher Scientific, T34077) were added, and plates were incubated for 30 min before starting live imaging on the high-content microscope Opera Phenix Plus by using the 63× water immersion objective with nine fields of view per well and z-stack of six planes (0.5 μm) with the environmental control enabled (5% CO_2_, 37°C and humidity). Images were acquired every 9 hours for four continuous days. The pFTAA signal was detected by using 405 nm/550 nm Ex/Em filters and tubulin at 647 nm/665 nm Ex/Em; BFP was also imaged as a nuclei marker (375 nm/450 nm Ex/Em, it is part of the hNGN2 plasmid). For experiments where we fixed cells after labeling with pFTAA, a similar approach was followed; however, the plates were maintained in the incubator before fixation with methanol. More than three independent inductions were performed for each line, and for each line, three to four technical replicates were included/induction.

Acquired images were analyzed in Harmony 5.1. Briefly, images were maximum projected, and basic flatfield correction was applied. Live cells were identified by the “find nuclei” (using the BFP channel) function, and dead cells were filtered out using different approaches such as nuclei size and intensity. The cytoplasm of living cells was then identified using the tubulin channel. “Find regions building block” was used to identify pFTAA-positive areas by using the absolute threshold option to make sure that we detected objects above background (600 to 700 was used as the lowest threshold). Objects where pFTAA was colocalized with dead cells were excluded from the analysis by filtering them out. Both pFTAA area and intensity were calculated and normalized to the number of live cells. Results are presented as integrated intensity, and data were plotted on GraphPad Prism.

### Quantitative PCR

iPSC samples for RNA extraction were collected by washing cells with DPBS and dissociating cells with either EDTA or ReLeSa, spinning down at 300*g* for 7 min at 4°C. Pellets were stored at −70°C. i^3^Ns were washed with PBS before adding fresh DPBS and lifting the monolayer by swirling the plate. Cells were collected and spun down at 300*g* for 7 min at 4°C, and pellets were stored at −70°C. RNA extraction was performed using ReliaPrep RNA Cell Miniprep System (Promega; Z6012) according to the manufacturer’s instructions. Reverse transcription was performed by using 300 ng RNA and LunaScript RT Supermix Kit (New England Biolabs; E3010) by following the manufacturer’s instructions. qPCR was performed using the Luna Universal qPCR Master Mix kit (New England Biolabs; M3003S), according to the manufacturer’s instructions, and reactions were carried out using the Lightcycler 480 II (Roche). Expression values were calculated in Excel using the Ct method, where glyceraldehyde-3-phosphate dehydrogenase (GAPDH) was used as a control.

### Semiquantitative PCR

semi-qPCR was conducted to quantify the ratio of 3R:4R mRNA. cDNA was used as a template, which amplified by primers (*24*) flanking exon 10 (forward 5′-AAGTCGCCGTCTTCCGCCAAG-3′; reverse 5′-GTCCAGGGACCCAATCTTCGA-3′). Q5 2× master mix (New England Biolabs; M0492S) was used for the amplification, and PCR products were run on a 2% agarose gel with 381– and 288–base pair fragments indicating 4R and 3R, respectively. Ratios were calculated as previously described (*62*), where ImageJ box plots and measure plots were used. Sum pixel intensity values were exported into Excel, and the percentage change was calculated by dividing each isoform value by the summed total intensity.

### Immunoblot analysis

All iPSC lines were differentiated into neurons on 6-well plates, and samples were collected at DIV7, 10, 14, 17, 21, 24, and 28 postinduction. Briefly, cells were washed with DPBS once before adding radioimmunoprecipitation assay (Thermo Fisher Scientific; 89900) buffer containing cOmplete protease inhibitor cocktail (Roche; 11697498001) and PhosSTOP (Roche; 11697498001). Lysates were incubated on ice for 20 min, centrifuged at 12,000*g* for 10 min at 4°C, and supernatants were collected. Samples for dephosphorylation assay were lysed in 50 mM tris (pH 7.6) and 0.15 M NaCl, cOmplete protease inhibitor cocktail. Protein concentration was determined by using Pierce BCA protein assay kit (Thermo Fisher Scientific; 23227) for all lysates. Equal amounts were loaded into 4 to 20% TGX Stain-Free gels (Bio-Rad; 5678094) or dephosphorylated with lambda-phosphatase (Santa Cruz Biotechnology; sc-200312A). Proteins were transferred on to low-fluorescent polyvinylidene difluoride membranes (Bio-Rad; 1620264) blocked with 5% milk in TBS-Tween (0.02% Tween). Primary antibodies were diluted in SuperBlock TBS-blocking buffer (Thermo Fisher Scientific; 37535) and incubated overnight at 4°C. Primary antibodies used included the following: mouse anti-Tau13 (N terminus tau, 1:1000, Santa Cruz Biotechnologies; sc-21796), rabbit anti-TauC (C terminus tau, 1:5000, Daco, A0024), mouse anti-AT8 (1:500, Invitrogen, MN1020), mouse anti-CP13 (1:500, Peter Davies), rat anti-tubulin (tyrosinated tubulin) (1:2000, Sigma-Aldrich; MAB1864), rabbit anti-PolyE tubulin (1:5000, gift from D. Villaroel), and anti–β-actin–fluorescein isothiocyanate (Millipore, F3022). Incubation with AT8 antibody was not compatible with the TGX Stain-Free gel for that reason, and 4 to 12% SDS–polyacrylamide gel electrophoresis gels (Invitrogen, NP0321BOX) were used. Secondary horseradish peroxidase–conjugated goat anti-rabbit or goat anti-mouse antibodies were diluted in 5% milk in TBS-Tween for 1 hour at room temperature. Chemiluminescence (Bio-Rad) was used for detection of immunoblotting, and bands were quantified by intensity using ImageLab (Bio-Rad). For the current study all blotted membranes were stripped by using PLUS Western blot Stripping Buffer (Thermo Fisher Scientific, 10016433) for 12 min before incubating with a new primary antibody.

### Sample preparation for seeding with i^3^N neurons

i^3^N neurons were placed into six-well plates, and samples for seeding were collected at DIV21 and DIV28. A whole 6-well plate was used for these experiments. Cells were washed with DPBS before adding fresh DPBS and lifting cells as a monolayer by swirling the plate. Cells were pelleted by centrifuging at 300*g* for 7 min at 4°C and stored at −70°C. Pellets were lysed with 50 mM tris (pH 7.6), 0.15 M NaCl, and cOmplete protease inhibitor cocktail. Samples were sonicated with a water bath sonicator (Qsonica Q800R3) at 4°C for 5 min at 65 amplitude (30 s on, 60 s off) before incubating on ice for 30 min. Samples were spun down at 300*g* for 5 min at 4°C, and supernatant was collected. Protein concentration was determined by using Pierce BCA protein assay kit.

### Biosensor cell culture method and seeding

Tau RD S305N-YFP human embryonic kidney (HEK) 293 biosensors and HEK293 Tau RD P301S FRET biosensor cells (American Type Culture Collection, CRL-3275) were cultured in DMEM (Thermo Fisher Scientific, catalog no. 41966-029) with 10% fetal bovine serum and 1% penicillin-streptomycin. Cells were plated at a density of 33,000 cells per well in a 96-well plate either PhenoPlates (Revvity) or standard tissue culture plates, in a volume of 130 μl of medium per well. After 18 hours, cells were transduced with seeds; 1.25 μl of Lipofectamine 2000 (Thermo Fisher Scientific, 11668019) was mixed with 8.75 μl Opti-MEM (Thermo Fisher Scientific, 31985062) and incubated at room temperature for 5 min before mixing with 18 μg of total protein of i^3^N total lysates. Complexes were incubated for 30 min at 37°C before transferring 20 μl to each well and further incubated with the cells for 72 hours. For each condition, three technical replicates were included. Three independent inductions were performed.

### Imaging analysis for the S305N biosensor line

In S305N biosensors, 1:10,000 Hoechst was added to the cells before imaging and incubated for 10 min. Plates were imaged on the high-content microscope Opera Phenix Plus using the 20× water immersion objective with 20 fields of view per well and a z-stack of eight planes (0.8 μm) with the environmental control enabled (5% CO_2_, 37°C, and humidity). Excitation wavelengths and emission filters were used as follows: YFP 488 nm/527 to 530 nm, Hoechst: 375 nm/435 to 480 nm. The acquired images were analyzed in Harmony 5.2. Briefly, images were loaded as maximum projection and basic flatfield correction was applied. Cells were segmented by using the find nuclei building block using the Hoechst channel, consequently, “find cytoplasm” was identified by using the watershed of the Hoechst channel. The YFP-positive tau inclusions were identified by using the “find spots” building block (method C) on the YFP channel. The analysis was exported, and the number of spots per nuclei was calculated.

### Flow cytometry analysis for P301S biosensor line

To quantify tau seeding in the P301S FRET biosensor, cells were harvested using 0.25% trypsin, fixed in 4% paraformaldehyde for 10 min, and resuspended in flow cytometry buffer (1× PBS with 1 mM EDTA). FRET flow cytometry was performed using an LSR Fortessa Flow Cytometer (BD Biosciences). FRET-positive cells were identified as previously described (*44*). In brief, single cells double-positive for CFP and YFP were gated, and FRET-positive events were quantified within this population. The percentage of FRET-positive cells was calculated as output metric. Data were analyzed using FCS Express v7 (De Novo Software) and GraphPad Prism.

### HiBiT lytic detection assay and viability assay

For the lytic assay, neurons were grown in white, Cellstar plates until DIV20 at 100 μl of maintenance media. Neurons were treated at the indicated time point. To assess the neuronal viability at the final time point GF-AFC substrate from MultiTox-Fluor Multiplex Cytotoxicity Assay (Promega, G9200) was used following the manufacturer’s protocol. Briefly, 20 μl from the 5× GF-AFC substrate was added into the cells, incubated for 30 min at 37°C before reading fluorescence (400Ex/505Em) at the FLUOstar Omega plate reader (BMG Labtech). Afterward, 120 μl of the Nano-Glo HiBiT Lytic Detection Reagent (Promega, N3040) was added directly to the cells and incubated for 10 min before recording luminescence on a FLUOstar Omega plate reader with 0.2 s integration time.

### Human tissue

Postmortem human brain tissue from an S305N carrier was provided by B. Boeve and D. Dickson from the Mayo ADRC (P30 AG062677)/ALLFTD (U19 AG063911). Ethical approval was granted by the Mayo Clinic.

### Immunohistochemistry

For human Formalin-fixed paraffin-embedded (FFPE) staining, 8-μm sections were deparaffinized in xylene and rehydrated using graded alcohols. Following pressure cooker pretreatment in citrate buffer for 10 min and endogenous per-oxidase quenching for 10 min, sections were blocked in 10% dried milk solution. Tissue sections were incubated with primary antibodies for 1 hour at room temperature, followed by biotinylated secondary incubation for 30 min at room temperature and avidin-biotin complex for 30 min. Color was developed with 3,3′-diaminobenzidine/H_2_O_2_. Sections were scanned on a NanoZoomer Digital Pathology C9600 (Hamamatsu Photonics).

### Statistical analysis

All statistical analysis was conducted at GraphPad Prism. *t* test was used to compare two groups and one-way analysis of variance (ANOVA) for three groups or more, with the appropriate post hoc test as stated in the figure legend. Two-way ANOVA was used for comparison between cell lines and time with the appropriate post hoc test.

## References

[1] J. Götz, G. Halliday, R. M. Nisbet, Molecular pathogenesis of the tauopathies. Annu. Rev. Pathol. 14, 239–261 (2019). 30355155 10.1146/annurev-pathmechdis-012418-012936

[2] C. A. Brunello, M. Merezhko, R. L. Uronen, H. J. Huttunen, Mechanisms of secretion and spreading of pathological tau protein. Cell. Mol. Life Sci. 77, 1721–1744 (2020). 31667556 10.1007/s00018-019-03349-1 PMC7190606

[3] S. C. Feinstein, L. Wilson, Inability of tau to properly regulate neuronal microtubule dynamics: A loss-of-function mechanism by which tau might mediate neuronal cell death. Biochim. Biophys. Acta 1739, 268–279 (2005). 15615645 10.1016/j.bbadis.2004.07.002

[4] M. Goedert, M. G. Spillantini, R. Jakes, D. Rutherford, R. A. Crowther, Multiple isoforms of human microtubule-associated protein tau: Sequences and localization in neurofibrillary tangles of Alzheimer’s disease. Neuron 3, 519–526 (1989). 2484340 10.1016/0896-6273(89)90210-9

[5] M. Goedert, R. Jakes, Expression of separate isoforms of human tau protein: Correlation with the tau pattern in brain and effects on tubulin polymerization. EMBO J. 9, 4225–4230 (1990). 2124967 10.1002/j.1460-2075.1990.tb07870.x PMC552204

[6] M. M. Hefti, K. Farrell, S. Kim, K. R. Bowles, M. E. Fowkes, T. Raj, J. F. Crary, High-resolution temporal and regional mapping of MAPT expression and splicing in human brain development. PLOS ONE 13, e0195771 (2018). 29634760 10.1371/journal.pone.0195771 PMC5892924

[7] D. Trabzuni, S. Wray, J. Vandrovcova, A. Ramasamy, R. Walker, C. Smith, C. Luk, J. R. Gibbs, A. Dillman, D. G. Hernandez, S. Arepalli, A. B. Singleton, M. R. Cookson, A. M. Pittman, R. de Silva, M. E. Weale, J. Hardy, M. Ryten, MAPT expression and splicing is differentially regulated by brain region: Relation to genotype and implication for tauopathies. Hum. Mol. Genet. 21, 4094–4103 (2012). 22723018 10.1093/hmg/dds238 PMC3428157

[8] J. E. Beevers, M. C. Lai, E. Collins, H. D. E. Booth, F. Zambon, L. Parkkinen, J. Vowles, S. A. Cowley, R. Wade-Martins, T. M. Caffrey, MAPT genetic variation and neuronal maturity alter isoform expression affecting axonal transport in iPSC-derived dopamine neurons. Stem Cell Rep. 9, 587–599 (2017). 10.1016/j.stemcr.2017.06.005 PMC5549835 28689993

[9] C. Conrad, J. Zhu, C. Conrad, D. Schoenfeld, Z. Fang, M. Ingelsson, S. Stamm, G. Church, B. T. Hyman, Single molecule profiling of tau gene expression in Alzheimer’s disease. J. Neurochem. 103, 1228–1236 (2007). 17727636 10.1111/j.1471-4159.2007.04857.x

[10] T. W. Rösler, A. Tayaranian Marvian, M. Brendel, N. P. Nykänen, M. Höllerhage, S. C. Schwarz, F. Hopfner, T. Koeglsperger, G. Respondek, K. Schweyer, J. Levin, V. L. Villemagne, H. Barthel, O. Sabri, U. Müller, W. G. Meissner, G. G. Kovacs, G. U. Höglinger, Four-repeat tauopathies. Prog. Neurobiol. 180, 101644 (2019). 31238088 10.1016/j.pneurobio.2019.101644

[11] M. Stamelou, G. Respondek, N. Giagkou, J. L. Whitwell, G. G. Kovacs, G. U. Höglinger, Evolving concepts in progressive supranuclear palsy and other 4-repeat tauopathies. Nat. Rev. Neurol. 17, 601–620 (2021). 34426686 10.1038/s41582-021-00541-5

[12] C. M. Karch, A. W. Kao, A. Karydas, K. Onanuga, R. Martinez, A. Argouarch, C. Wang, C. Huang, P. D. Sohn, K. R. Bowles, S. Spina, M. C. Silva, J. A. Marsh, S. Hsu, D. A. Pugh, N. Ghoshal, J. Norton, Y. Huang, S. E. Lee, W. W. Seeley, P. Theofilas, L. T. Grinberg, F. Moreno, K. McIlroy, B. F. Boeve, N. J. Cairns, J. F. Crary, S. J. Haggarty, J. K. Ichida, K. S. Kosik, B. L. Miller, L. Gan, A. M. Goate, S. Temple, C. Alquezar, K. Bowles, D. Butler, J. F. Crary, L. Gan, A. M. Goate, S. J. Haggarty, I. Hernandez, V. Hennes, C. Huang, J. K. Ichida, M. Kampmann, A. W. Kao, C. M. Karch, A. Karydas, K. S. Kosik, R. Martinez, K. Onanuga, M. C. Silva, S. Temple, C. Wang, A comprehensive resource for induced pluripotent stem cells from patients with primary tauopathies. Stem Cell Rep. 13, 939–955 (2019). 10.1016/j.stemcr.2019.09.006 PMC6895712 31631020

[13] S. Mahali, C. Karch, Defective proteostasis in patient-derived iPSC-astrocytes and neurons carrying a MAPT IVS10+16 mutation. Alzheimers Dement. 17, e058727 (2021).

[14] M. C. Silva, C. Cheng, W. Mair, S. Almeida, H. Fong, M. H. U. Biswas, Z. Zhang, Y. Huang, S. Temple, G. Coppola, D. H. Geschwind, A. Karydas, B. L. Miller, K. S. Kosik, F. B. Gao, J. A. Steen, S. J. Haggarty, Human iPSC-derived neuronal model of Tau-A152T frontotemporal dementia reveals tau-mediated mechanisms of neuronal vulnerability. Stem Cell Rep. 7, 325–340 (2016). 10.1016/j.stemcr.2016.08.001 PMC5032560 27594585

[15] S. Wray, Modeling tau pathology in human stem cell derived neurons. Brain Pathol. 27, 525–529 (2017). 28585382 10.1111/bpa.12521 PMC8029337

[16] A. E. Handel, S. Chintawar, T. Lalic, E. Whiteley, J. Vowles, A. Giustacchini, K. Argoud, P. Sopp, M. Nakanishi, R. Bowden, S. Cowley, S. Newey, C. Akerman, C. P. Ponting, M. Z. Cader, Assessing similarity to primary tissue and cortical layer identity in induced pluripotent stem cell-derived cortical neurons through single-cell transcriptomics. Hum. Mol. Genet. 25, 989–1000 (2016). 26740550 10.1093/hmg/ddv637 PMC4754051

[17] T. Sposito, E. Preza, C. J. Mahoney, N. Setó-Salvia, N. S. Ryan, H. R. Morris, C. Arber, M. J. Devine, H. Houlden, T. T. Warner, T. J. Bushell, M. Zagnoni, T. Kunath, F. J. Livesey, N. C. Fox, M. N. Rossor, J. Hardy, S. Wray, Developmental regulation of tau splicing is disrupted in stem cell-derived neurons from frontotemporal dementia patients with the 10 + 16 splice-site mutation in MAPT. Hum. Mol. Genet. 24, 5260–5269 (2015). 26136155 10.1093/hmg/ddv246 PMC4550814

[18] A. Verheyen, A. Diels, J. Reumers, K. Van Hoorde, I. Van den Wyngaert, C. van Outryve d’Ydewalle, A. De Bondt, J. Kuijlaars, L. De Muynck, R. De Hoogt, A. Bretteville, S. Jaensch, A. Buist, A. Cabrera-Socorro, S. Wray, A. Ebneth, P. Roevens, I. Royaux, P. J. Peeters, Genetically engineered iPSC-derived FTDP-17 MAPT neurons display mutation-specific neurodegenerative and neurodevelopmental phenotypes. Stem Cell Rep. 11, 363–379 (2018). 10.1016/j.stemcr.2018.06.022 PMC6093179 30057263

[19] L. Miguel, A. Rovelet-Lecrux, M. Feyeux, T. Frebourg, P. Nassoy, D. Campion, M. Lecourtois, Detection of all adult Tau isoforms in a 3D culture model of iPSC-derived neurons. Stem Cell Res. 40, 101541 (2019). 31522011 10.1016/j.scr.2019.101541

[20] J. A. García-León, A. Cabrera-Socorro, K. Eggermont, A. Swijsen, J. Terryn, R. Fazal, F. Nami, L. Ordovás, A. Quiles, F. Lluis, L. Serneels, K. Wierda, A. Sierksma, M. Kreir, F. Pestana, P. Van Damme, B. De Strooper, L. Thorrez, A. Ebneth, C. M. Verfaillie, Generation of a human induced pluripotent stem cell–based model for tauopathies combining three microtubule-associated protein TAU mutations which displays several phenotypes linked to neurodegeneration. Alzheimers Dement. 14, 1261–1280 (2018). 30036493 10.1016/j.jalz.2018.05.007

[21] C. Parra Bravo, A. M. Giani, J. Madero-Perez, Z. Zhao, Y. Wan, A. J. Samelson, M. Y. Wong, A. Evangelisti, E. Cordes, L. Fan, P. Ye, D. Zhu, T. Pozner, M. Mercedes, T. Patel, A. Yarahmady, G. K. Carling, F. H. Sterky, V. M. Y. Lee, E. B. Lee, M. DeTure, D. W. Dickson, M. Sharma, S.-A. Mok, W. Luo, M. Zhao, M. Kampmann, S. Gong, L. Gan, Human iPSC 4R tauopathy model uncovers modifiers of tau propagation. Cell 187, 2446–2464.e22 (2024). 38582079 10.1016/j.cell.2024.03.015 PMC11365117

[22] A. Dannert, N. Schulz, J. Klimmt, L. Knez, B. Groschup, C. C. Gonçalves, C. Carraro, M. Schifferer, M. Brendel, D. Paquet, A human iPSC model of tauopathies engineered for 4R tau isoform expression endogenously develops late-stage neuronal tau pathology. Sci. Transl. Med. 18, eadu9845 (2026). 41950304 10.1126/scitranslmed.adu9845

[23] L. S. Capano, C. Sato, E. Ficulle, A. Yu, K. Horie, J. S. Kwon, K. F. Burbach, N. R. Barthélemy, S. G. Fox, C. M. Karch, R. J. Bateman, H. Houlden, R. I. Morimoto, D. M. Holtzman, K. E. Duff, A. S. Yoo, Recapitulation of endogenous 4R tau expression and formation of insoluble tau in directly reprogrammed human neurons. Cell Stem Cell 29, 918–932.e8 (2022). 35659876 10.1016/j.stem.2022.04.018 PMC9176216

[24] S. H. Choi, Y. H. Kim, M. Hebisch, C. Sliwinski, S. Lee, C. D’Avanzo, H. Chen, B. Hooli, C. Asselin, J. Muffat, J. B. Klee, C. Zhang, B. J. Wainger, M. Peitz, D. M. Kovacs, C. J. Woolf, S. L. Wagner, R. E. Tanzi, D. Y. Kim, A three-dimensional human neural cell culture model of Alzheimer’s disease. Nature 515, 274–278 (2014). 25307057 10.1038/nature13800 PMC4366007

[25] M. Hutton, C. L. Lendon, P. Rizzu, M. Baker, S. Froelich, H. Houlden, S. Pickering-Brown, S. Chakraverty, A. Isaacs, A. Grover, J. Hackett, J. Adamson, S. Lincoln, D. Dickson, P. Davies, R. C. Petersen, M. Stevens, E. de Graaff, E. Wauters, J. van Baren, M. Hillebrand, M. Joosse, J. M. Kwon, P. Nowotny, L. K. Che, J. Norton, J. C. Morris, L. A. Reed, J. Trojanowski, H. Basun, L. Lannfelt, M. Neystat, S. Fahn, F. Dark, T. Tannenberg, P. R. Dodd, N. Hayward, J. B. Kwok, P. R. Schofield, A. Andreadis, J. Snowden, D. Craufurd, D. Neary, F. Owen, B. A. Oostra, J. Hardy, A. Goate, J. van Swieten, D. Mann, T. Lynch, P. Heutink, Association of missense and 5′-splice-site mutations in tau with the inherited dementia FTDP-17. Nature 393, 702–705 (1998). 9641683 10.1038/31508

[26] M. Hasegawa, M. J. Smith, M. Iijima, T. Tabira, M. Goedert, FTDP-17 mutations N279K and S305N in tau produce increased splicing of exon 10. FEBS Lett. 443, 93–96 (1999). 9989582 10.1016/s0014-5793(98)01696-2

[27] M. G. Spillantini, M. Goedert, Tau pathology and neurodegeneration. Lancet Neurol. 12, 609–622 (2013). 23684085 10.1016/S1474-4422(13)70090-5

[28] B. F. Boeve, I. W. Tremont-Lukats, A. J. Waclawik, J. R. Murrell, B. Hermann, C. R. Jack Jr., M. M. Shiung, G. E. Smith, A. R. Nair, N. Lindor, V. Koppikar, B. Ghetti, Longitudinal characterization of two siblings with frontotemporal dementia and parkinsonism linked to chromosome 17 associated with the S305N tau mutation. Brain 128, 752–772 (2005). 15615814 10.1093/brain/awh356

[29] K. Kobayashi, T. Kidani, H. Ujike, M. Hayashi, T. Ishihara, K. Miyazu, S. Kuroda, Y. Koshino, Another phenotype of frontotemporal dementia and parkinsonism linked to chromosome-17 (FTDP-17) with a missense mutation of S305N closely resembling Pick’s disease. J. Neurol. 250, 990–992 (2003). 12928922 10.1007/s00415-003-1137-6

[30] K. R. Bowles, C. Pedicone, D. A. Pugh, L.-M. Oja, F. H. Sousa, L. K. Keavey, B. Fulton-Howard, S. A. Weitzman, Y. Liu, J. L. Chen, M. D. Disney, A. M. Goate, Development of MAPT S305 mutation human iPSC lines exhibiting elevated 4R tau expression and functional alterations in neurons and astrocytes. Cell Rep. 43, 115013 (2024). 39602304 10.1016/j.celrep.2024.115013 PMC13478741

[31] N. Watamura, M. S. Foiani, S. Bez, M. Bourdenx, A. Santambrogio, C. Frodsham, E. Camporesi, G. Brinkmalm, H. Zetterberg, S. Patel, N. Kamano, M. Takahashi, J. Rueda-Carrasco, L. Katsouri, S. Fowler, E. Turkes, S. Hashimoto, H. Sasaguri, T. Saito, A. S. Islam, S. Benner, T. Endo, K. Kobayashi, C. Ishida, M. Vendruscolo, M. Yamada, K. E. Duff, T. C. Saido, In vivo hyperphosphorylation of tau is associated with synaptic loss and behavioral abnormalities in the absence of tau seeds. Nat. Neurosci. 28, 293–307 (2025). 39719507 10.1038/s41593-024-01829-7 PMC11802456

[32] J. L. Chen, W. N. Moss, A. Spencer, P. Zhang, J. L. Childs-Disney, M. D. Disney, The RNA encoding the microtubule-associated protein tau has extensive structure that affects its biology. PLOS ONE 14, e0219210 (2019). 31291322 10.1371/journal.pone.0219210 PMC6619747

[33] M. Iijima, T. Tabira, P. Poorkaj, G. D. Schellenberg, J. Q. Trojanowski, V. M. Lee, M. L. Schmidt, K. Takahashi, T. Nabika, T. Matsumoto, Y. Yamashita, S. Yoshioka, H. Ishino, A distinct familial presenile dementia with a novel missense mutation in the tau gene. Neuroreport 10, 497–501 (1999). 10208578 10.1097/00001756-199902250-00010

[34] M. G. Spillantini, J. R. Murrell, M. Goedert, M. R. Farlow, A. Klug, B. Ghetti, Mutation in the tau gene in familial multiple system tauopathy with presenile dementia. Proc. Natl. Acad. Sci. U.S.A. 95, 7737–7741 (1998). 9636220 10.1073/pnas.95.13.7737 PMC22742

[35] C. B. Pantazis, A. Yang, E. Lara, J. A. McDonough, C. Blauwendraat, L. Peng, H. Oguro, J. Kanaujiya, J. Zou, D. Sebesta, G. Pratt, E. Cross, J. Blockwick, P. Buxton, L. Kinner-Bibeau, C. Medura, C. Tompkins, S. Hughes, M. Santiana, F. Faghri, M. A. Nalls, D. Vitale, S. Ballard, Y. A. Qi, D. M. Ramos, K. M. Anderson, J. Stadler, P. Narayan, J. Papademetriou, L. Reilly, M. P. Nelson, S. Aggarwal, L. U. Rosen, P. Kirwan, V. Pisupati, S. L. Coon, S. W. Scholz, T. Priebe, M. Öttl, J. Dong, M. Meijer, L. J. M. Janssen, V. S. Lourenco, R. van der Kant, D. Crusius, D. Paquet, A.-C. Raulin, G. Bu, A. Held, B. J. Wainger, R. M. C. Gabriele, J. M. Casey, S. Wray, D. Abu-Bonsrah, C. L. Parish, M. S. Beccari, D. W. Cleveland, E. Li, I. V. L. Rose, M. Kampmann, C. Calatayud Aristoy, P. Verstreken, L. Heinrich, M. Y. Chen, B. Schüle, D. Dou, E. L. F. Holzbaur, M. C. Zanellati, R. Basundra, M. Deshmukh, S. Cohen, R. Khanna, M. Raman, Z. S. Nevin, M. Matia, J. Van Lent, V. Timmerman, B. R. Conklin, K. Johnson Chase, K. Zhang, S. Funes, D. A. Bosco, L. Erlebach, M. Welzer, D. Kronenberg-Versteeg, G. Lyu, E. Arenas, E. Coccia, L. Sarrafha, T. Ahfeldt, J. C. Marioni, W. C. Skarnes, M. R. Cookson, M. E. Ward, F. T. Merkle, A reference human induced pluripotent stem cell line for large-scale collaborative studies. Cell Stem Cell 29, 1685–1702.e22 (2022). 36459969 10.1016/j.stem.2022.11.004 PMC9782786

[36] M. S. Fernandopulle, R. Prestil, C. Grunseich, C. Wang, L. Gan, M. E. Ward, Transcription factor-mediated differentiation of human iPSCs into neurons. Curr. Protoc. Cell Biol. 79, e51 (2018). 29924488 10.1002/cpcb.51 PMC6993937

[37] W. M. Seo, J. Yoon, J.-H. Lee, Y. Lee, H. Lee, D. Geum, W. Sun, M.-R. Song, Modeling axonal regeneration by changing cytoskeletal dynamics in stem cell-derived motor nerve organoids. Sci. Rep. 12, 2082 (2022). 35136073 10.1038/s41598-022-05645-6 PMC8827082

[38] H. Faris, M. Almasieh, L. A. Levin, Axonal degeneration induces distinct patterns of phosphatidylserine and phosphatidylethanolamine externalization. Cell Death Discov. 7, 247 (2021). 34535640 10.1038/s41420-021-00641-7 PMC8448818

[39] D. Chu, X. Yang, J. Wang, Y. Zhou, J. H. Gu, J. Miao, F. Wu, F. Liu, Tau truncation in the pathogenesis of Alzheimer’s disease: A narrative review. Neural Regen. Res. 19, 1221–1232 (2024). 37905868 10.4103/1673-5374.385853 PMC11467920

[40] J. P. Quinn, N. J. Corbett, K. A. B. Kellett, N. M. Hooper, Tau proteolysis in the pathogenesis of tauopathies: Neurotoxic fragments and novel biomarkers. J. Alzheimer’s Dis 63, 13–33 (2018). 29630551 10.3233/JAD-170959 PMC5900574

[41] K. Kobayashi, M. Hayashi, T. Kidani, H. Ujike, M. Iijima, T. Ishihara, H. Nakano, K. Sugimori, M. Shimazaki, S. Kuroda, Y. Koshino, Pick’s disease pathology of a missense mutation of S305N of frontotemporal dementia and parkinsonism linked to chromosome 17: Another phenotype of S305N. Dement. Geriatr. Cogn. Disord. 17, 293–297 (2004). 15178939 10.1159/000077157

[42] J. L. Whitwell, C. R. Jack Jr., B. F. Boeve, M. L. Senjem, M. Baker, R. J. Ivnik, D. S. Knopman, Z. K. Wszolek, R. C. Petersen, R. Rademakers, K. A. Josephs, Atrophy patterns in IVS10+16, IVS10+3, N279K, S305N, P301L, and V337M MAPT mutations. Neurology 73, 1058–1065 (2009). 19786698 10.1212/WNL.0b013e3181b9c8b9 PMC2754325

[43] S. M. Ward, D. S. Himmelstein, J. K. Lancia, Y. Fu, K. R. Patterson, L. I. Binder, TOC1: Characterization of a selective oligomeric tau antibody. J. Alzheimer's Dis 37, 593–602 (2013). 23979027 10.3233/JAD-131235 PMC4791958

[44] B. B. Holmes, J. L. Furman, T. E. Mahan, T. R. Yamasaki, H. Mirbaha, W. C. Eades, L. Belaygorod, N. J. Cairns, D. M. Holtzman, M. I. Diamond, Proteopathic tau seeding predicts tauopathy in vivo. Proc. Natl. Acad. Sci. U.S.A. 111, E4376–E4385 (2014). 25261551 10.1073/pnas.1411649111 PMC4205609

[45] J. Brelstaff, B. Ossola, J. J. Neher, T. Klingstedt, K. P. R. Nilsson, M. Goedert, M. G. Spillantini, A. M. Tolkovsky, The fluorescent pentameric oligothiophene pFTAA identifies filamentous tau in live neurons cultured from adult P301S tau mice. Front. Neurosci. 9, 184 (2015). 26074756 10.3389/fnins.2015.00184 PMC4448042

[46] J. Brelstaff, M. G. Spillantini, A. M. Tolkovsky, pFTAA: A high affinity oligothiophene probe that detects filamentous tau: in vivo: and in cultured neurons. Neural Regen. Res. 10, 1746–1747 (2015). 26807101 10.4103/1673-5374.165298 PMC4705778

[47] G. Fu, S. Yan, C. J. Khoo, V. C. Chao, Z. Liu, M. Mukhi, R. Hervas, X. D. Li, S.-C. Ti, Integrated regulation of tubulin tyrosination and microtubule stability by human α-tubulin isotypes. Cell Rep. 42, 112653 (2023). 37379209 10.1016/j.celrep.2023.112653

[48] C. Sanyal, N. Pietsch, S. Ramirez Rios, L. Peris, L. Carrier, M.-J. Moutin, The detyrosination/re-tyrosination cycle of tubulin and its role and dysfunction in neurons and cardiomyocytes. Semin. Cell Dev. Biol. 137, 46–62 (2023). 34924330 10.1016/j.semcdb.2021.12.006

[49] M. Genova, L. Grycova, V. Puttrich, M. M. Magiera, Z. Lansky, C. Janke, M. Braun, Tubulin polyglutamylation differentially regulates microtubule-interacting proteins. EMBO J. 42, e112101 (2023). 36636822 10.15252/embj.2022112101 PMC9975938

[50] L. Hosseini-Gerami, E. Ficulle, N. Humphryes-Kirilov, D. C. Airey, J. Scherschel, S. Kananathan, B. J. Eastwood, S. Bose, D. A. Collier, E. Laing, D. Evans, H. Broughton, A. Bender, Mechanism of action deconvolution of the small-molecule pathological tau aggregation inhibitor Anle138b. Alzheimer’s Res. Ther. 15, 52 (2023). 36918909 10.1186/s13195-023-01182-0 PMC10012450

[51] C. Langworth-Green, S. Patel, Z. Jaunmuktane, E. Jabbari, H. Morris, M. Thom, A. Lees, J. Hardy, M. Zandi, K. Duff, Chronic effects of inflammation on tauopathies. Lancet Neurol. 22, 430–442 (2023). 37059510 10.1016/S1474-4422(23)00038-8

[52] M. Regalado-Reyes, D. Furcila, F. Hernández, J. Ávila, J. DeFelipe, G. León-Espinosa, Phospho-tau changes in the human CA1 during Alzheimer’s disease progression. J. Alzheimer’s Dis 69, 277–288 (2019). 30958368 10.3233/JAD-181263 PMC6598029

[53] H. Ksiezak-Reding, D. He, W. Gordon-Krajcer, Y. Kress, S. Lee, D. W. Dickson, Induction of Alzheimer-specific tau epitope AT100 in apoptotic human fetal astrocytes. Cell Motil. 47, 236–252 (2000). 10.1002/1097-0169(200011)47:3<236::AID-CM6>3.0.CO;2-K 11056524

[54] A. D. Alonso, L. S. Cohen, C. Corbo, V. Morozova, A. ElIdrissi, G. Phillips, F. E. Kleiman, Hyperphosphorylation of Tau associates with changes in its function beyond microtubule stability. Front. Cell. Neurosci. 12, 338 (2018). 30356756 10.3389/fncel.2018.00338 PMC6189415

[55] A. D. Alonso, J. Di Clerico, B. Li, C. P. Corbo, M. E. Alaniz, I. Grundke-Iqbal, K. Iqbal, Phosphorylation of tau at Thr212, Thr231, and Ser262 combined causes neurodegeneration. J. Biol. Chem. 285, 30851–30860 (2010). 20663882 10.1074/jbc.M110.110957 PMC2945578

[56] H. Yoshida, M. Goedert, Sequential phosphorylation of tau protein by cAMP-dependent protein kinase and SAPK4/p38delta or JNK2 in the presence of heparin generates the AT100 epitope. J. Neurochem. 99, 154–164 (2006). 16987243 10.1111/j.1471-4159.2006.04052.x

[57] Y. Akwa, E. Gondard, A. Mann, E. Capetillo-Zarate, E. Alberdi, C. Matute, S. Marty, T. Vaccari, A. M. Lozano, E. E. Baulieu, D. Tampellini, Synaptic activity protects against AD and FTD-like pathology via autophagic-lysosomal degradation. Mol. Psychiatry 23, 1530–1540 (2018). 28696431 10.1038/mp.2017.142 PMC5641448

[58] T. Hamano, T. F. Gendron, E. Causevic, S. H. Yen, W. L. Lin, C. Isidoro, M. Deture, L. W. Ko, Autophagic-lysosomal perturbation enhances tau aggregation in transfectants with induced wild-type tau expression. Eur. J. Neurosci. 27, 1119–1130 (2008). 18294209 10.1111/j.1460-9568.2008.06084.x

[59] B. Hempen, J. P. Brion, Reduction of acetylated alpha-tubulin immunoreactivity in neurofibrillary tangle-bearing neurons in Alzheimer’s disease. J. Neuropathol. Exp. Neurol. 55, 964–972 (1996). 8800092 10.1097/00005072-199609000-00003

[60] C. Cook, Y. Carlomagno, T. F. Gendron, J. Dunmore, K. Scheffel, C. Stetler, M. Davis, D. Dickson, M. Jarpe, M. DeTure, L. Petrucelli, Acetylation of the KXGS motifs in tau is a critical determinant in modulation of tau aggregation and clearance. Hum. Mol. Genet. 23, 104–116 (2013). 23962722 10.1093/hmg/ddt402 PMC3857946

[61] C. Sato, N. R. Barthélemy, K. G. Mawuenyega, B. W. Patterson, B. A. Gordon, J. Jockel-Balsarotti, M. Sullivan, M. J. Crisp, T. Kasten, K. M. Kirmess, N. M. Kanaan, K. E. Yarasheski, A. Baker-Nigh, T. L. S. Benzinger, T. M. Miller, C. M. Karch, R. J. Bateman, Tau kinetics in neurons and the human central nervous system. Neuron 97, 1284–1298.e7 (2018). 29566794 10.1016/j.neuron.2018.02.015 PMC6137722

[62] J. F. Antiabong, M. G. Ngoepe, A. S. Abechi, Semi-quantitative digital analysis of polymerase chain reaction-electrophoresis gel: Potential applications in low-income veterinary laboratories. Vet. World 9, 935–939 (2016). 27733792 10.14202/vetworld.2016.935-939 PMC5057030

