# Supplementary material for: Scalable human neuronal models of tauopathy producing endogenous seed-competent 4R tau
Source: Sci Adv. 2026 Jul 31;12(31):eaeg1445. doi: 10.1126/sciadv.aeg1445 (PMC13426404; doi:10.1126/sciadv.aeg1445)
Supplement: Supplementary file 1 — Figs. S1 to S8 Table S1 [file sciadv.aeg1445_sm.pdf]

Science Advances
AAAS

Supplementary Materials for

Scalable human neuronal models of tauopathy producing endogenous seed-competent 4R tau

Eliona Tsefou *et al.*

Corresponding author: Eliona Tsefou, e.tsefou@ucl.ac.uk; Karen E. Duff, k.duff@ucl.ac.uk

Sci. Adv. **12**, eaeg1445 (2026)
DOI: 10.1126/sciadv.aeg1445

This PDF file includes:

Figs. S1 to S8
Table S1

Supplementary Figures:

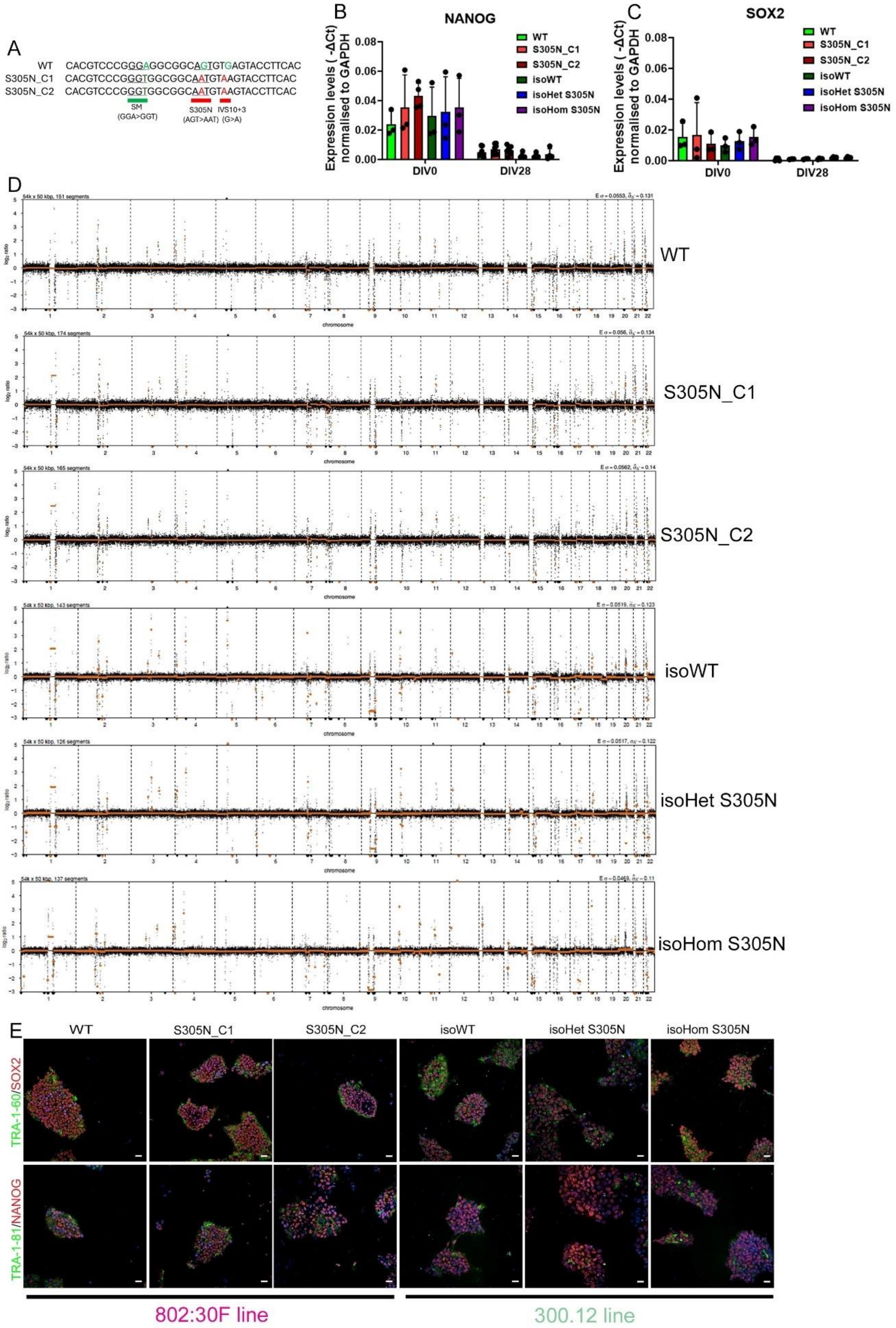

Fig S1. Characterisation of iPSCs with S305N tau mutations related to Fig. 1. (A) Schematic representation of the inserted mutations in the 802:30F iPSC line used to generate the S305N_C1 and S305N_C2 clones. (B–C) mRNA expression levels of the pluripotency markers NANOG (B) and SOX2 (C) in all lines at DIV0 (iPSC stage) and DIV28 (i^3N neurons). Data are presented as mean ± SD, n = 3–4 independent differentiations per presented neuronal line. (D) Low-coverage whole-genome sequencing revealed no chromosomal abnormalities in any of the lines following hNGN2 insertion. (E) Immunostaining for pluripotency markers TRA-1-60/SOX2 and TRA-1-81/NANOG confirmed the maintenance of pluripotency in all iPSC lines after hNGN2 integration.

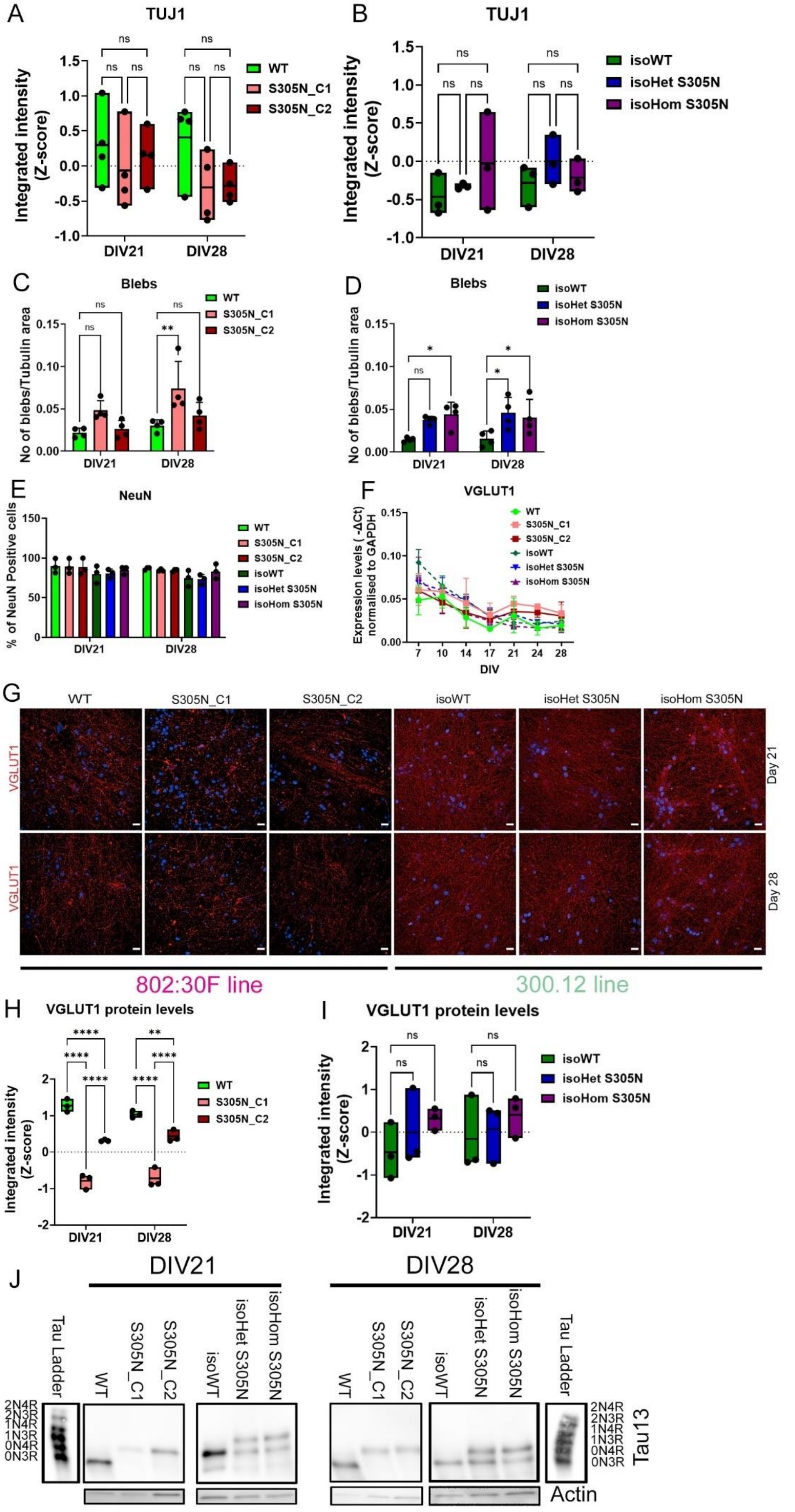

A
TUJ1
Integrated intensity (Z-score)
DIV21
DIV28
WT
S305N_C1
S305N_C2
B
TUJ1
isoWT
isoHet S305N
isoHom S305N
C
Blebs
No of blebs/Tubulin area
D
Blebs
E
NeuN
% of NeuN Positive cells
F
VGLUT1
Expression levels (-ΔCt) normalised to GAPDH
DIV
G
WT
S305N_C1
S305N_C2
isoWT
isoHet S305N
isoHom S305N
VGLUT1
Day 21
Day 28
802:30F line
300.12 line
H
VGLUT1 protein levels
I
VGLUT1 protein levels
J
DIV21
DIV28
Tau Ladder
2N4R
2N3R
1N4R
1N3R
0N4R
0N3R
Tau13
Actin

Fig. S2. Characterising S305N i^{3}N neurons related to Fig. 1. (A) Expression levels of TUJ1 in WT and S305N_C1 and C2 neurons at DIV21 and DIV28 measured in all imaged area (normalized to cell number). (B) Expression levels of TUJ1 in isoWT, isoHet, and isoHom S305N neurons at DIV21 and DIV28 measured in all imaged area (normalized to cell number). (C) Quantification of the number of blebs per TUJ1-positive area in WT and S305N_C1 and C2 neurons at DIV21 and DIV28. (D) Quantification of blebs per TUJ1-positive area in isoWT, isoHet, and isoHom S305N neurons at DIV21 and DIV28. (E) Percentage of NeuN-positive neurons in all lines at DIV21 and DIV28. (F) VGLUT1 mRNA levels across all lines over time. (G) Representative images of VGLUT1 staining in all neuronal lines at DIV21 and DIV28 (cells fixed with 4% PFA). (H) Quantification of VGLUT1 expression in WT and S305N_C1 and C2 neurons at DIV21 and DIV28 measured in all imaged area (normalized to cell number). (I) Quantification of VGLUT1 expression in isoWT, isoHet, and isoHom S305N neurons at DIV21 and DIV28 measured in all imaged area (normalized to cell number). (J) Representative immunoblots from the dephosphorylation assay for tau in all neuronal lines at DIV21 and DIV28. Tau13 was used to detect different tau isoforms. Statistical analysis for all panels was performed using two-way ANOVA followed by Tukey's post-hoc test. Data are presented as mean ± SD. Sample sizes for A-F, H, I: n = 3–5 independent differentiations per presented neuronal line. Significance: $^{**}p < 0.01$ $^{****}p < 0.0001$.

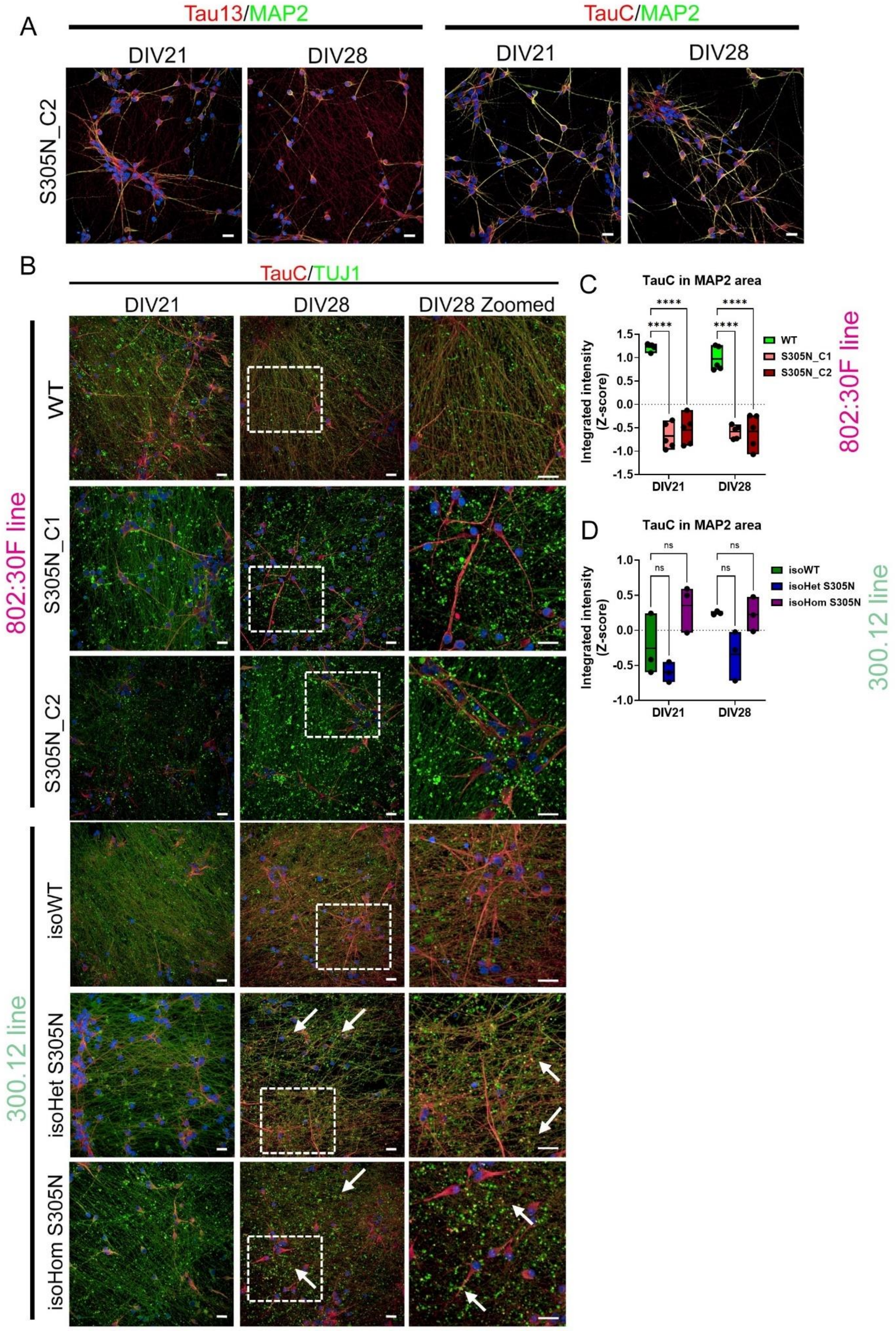
A
Tau13/MAP2
TauC/MAP2
DIV21
DIV28
DIV21
DIV28
S305N_C2
B
TauC/TUJ1
DIV21
DIV28
DIV28 Zoomed
802:30F line
WT
S305N_C1
S305N_C2
300.12 line
isoWT
isoHet S305N
isoHom S305N
C
TauC in MAP2 area
Integrated intensity (Z-score)
****
****
****
****
1.5
1.0
0.5
0.0
-0.5
-1.0
-1.5
DIV21
DIV28
WT
S305N_C1
S305N_C2
802:30F line
D
TauC in MAP2 area
Integrated intensity (Z-score)
ns
ns
ns
ns
1.0
0.5
0.0
-0.5
-1.0
DIV21
DIV28
isoWT
isoHet S305N
isoHom S305N
300.12 line

Fig. S3. Distribution of total tau in S305N i^{3}N neurons related to Fig. 2. (A) Representative images of DIV21 and DIV28 S305N_C2 i^{3}N neurons double-labelled with MAP2 and total tau antibodies: Tau13 (left) and TauC (right). (B) Representative images showing TauC co-labelled with TUJ1 across all neuronal lines. The enlarged image highlights colocalization of TauC-positive blebs with TUJ1-positive axonal blebs. Quantification of TauC in MAP2 positive area in the (C) 802:30F and (D) 300.12 lines at DIV21 and DIV28. Statistical analysis was performed using two-way ANOVA followed by Tukey's post-hoc test (mean ± SD, n =3 independent differentiations per presented neuronal line, ****, $p<0.0001$).

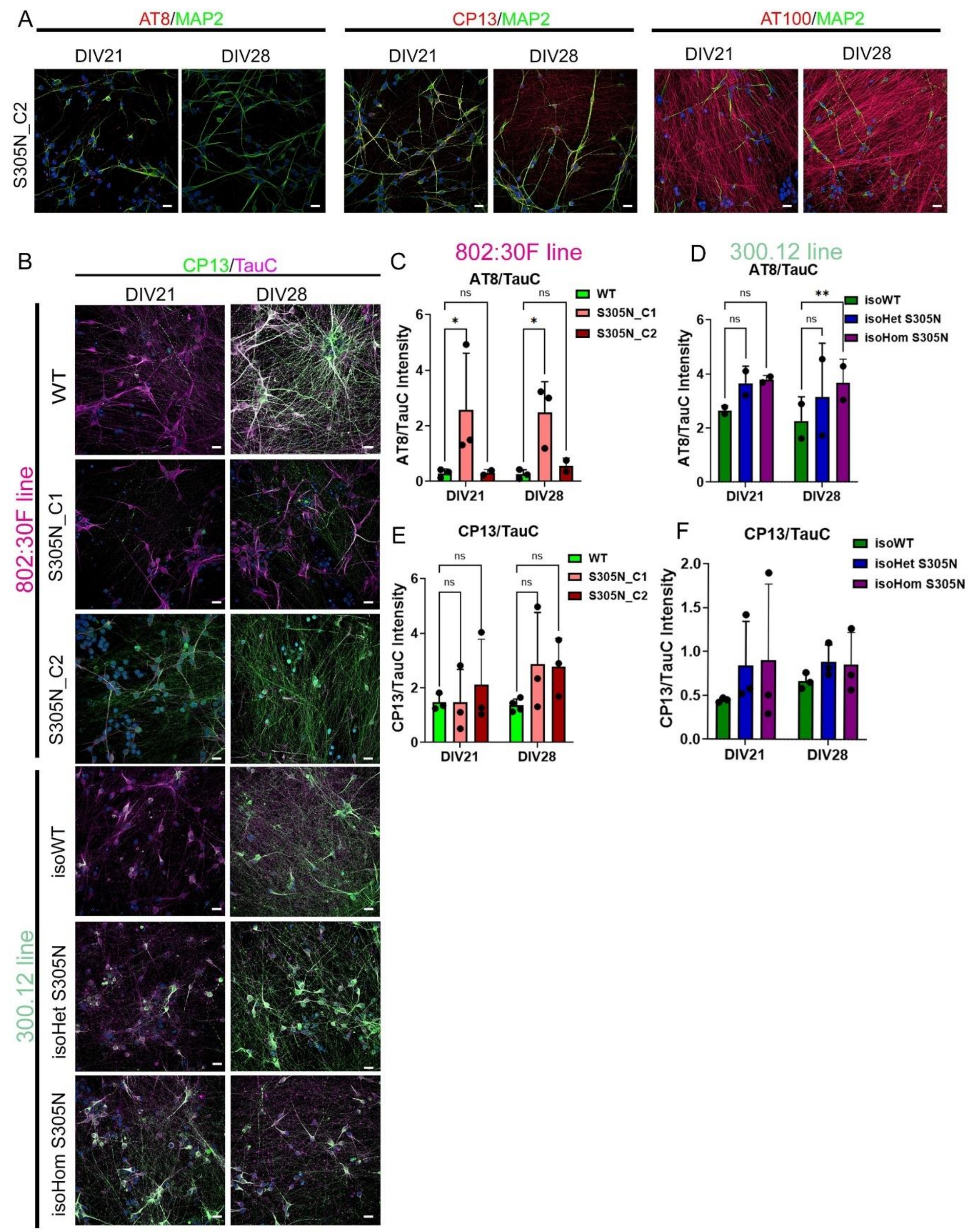

Fig. S4. Distribution of phosphorylated tau in S305N i^{3}N neurons related to Fig. 3. (A) Representative images of DIV21 and DIV28 S305N_C2 i^{3}N neurons double-labelled with MAP2 and phosphorylated tau antibodies: AT8 (left), CP13 (middle), and AT100 (right). (B) Representative images showing co-staining of CP13 and TauC across all neuronal lines. Quantification of AT8 immunostaining normalized to TauC in (C) 802:307 and (D) 300.12 lines at DIV21 and DIV28. Statistical analysis was performed using two-way ANOVA followed by Tukey's post-hoc test (mean ± SD, n = 2–3 independent neuronal differentiations).

Quantification of CP13 immunostaining normalized to TauC in (E) 802:307 and (F) 300.12 lines at DIV21 and DIV28. Statistical analysis was performed using two-way ANOVA followed by Tukey's post-hoc test (mean ± SD, n = 3–4 independent differentiations per presented neuronal line, *, $p<0.05$; **, $p<0.01$).

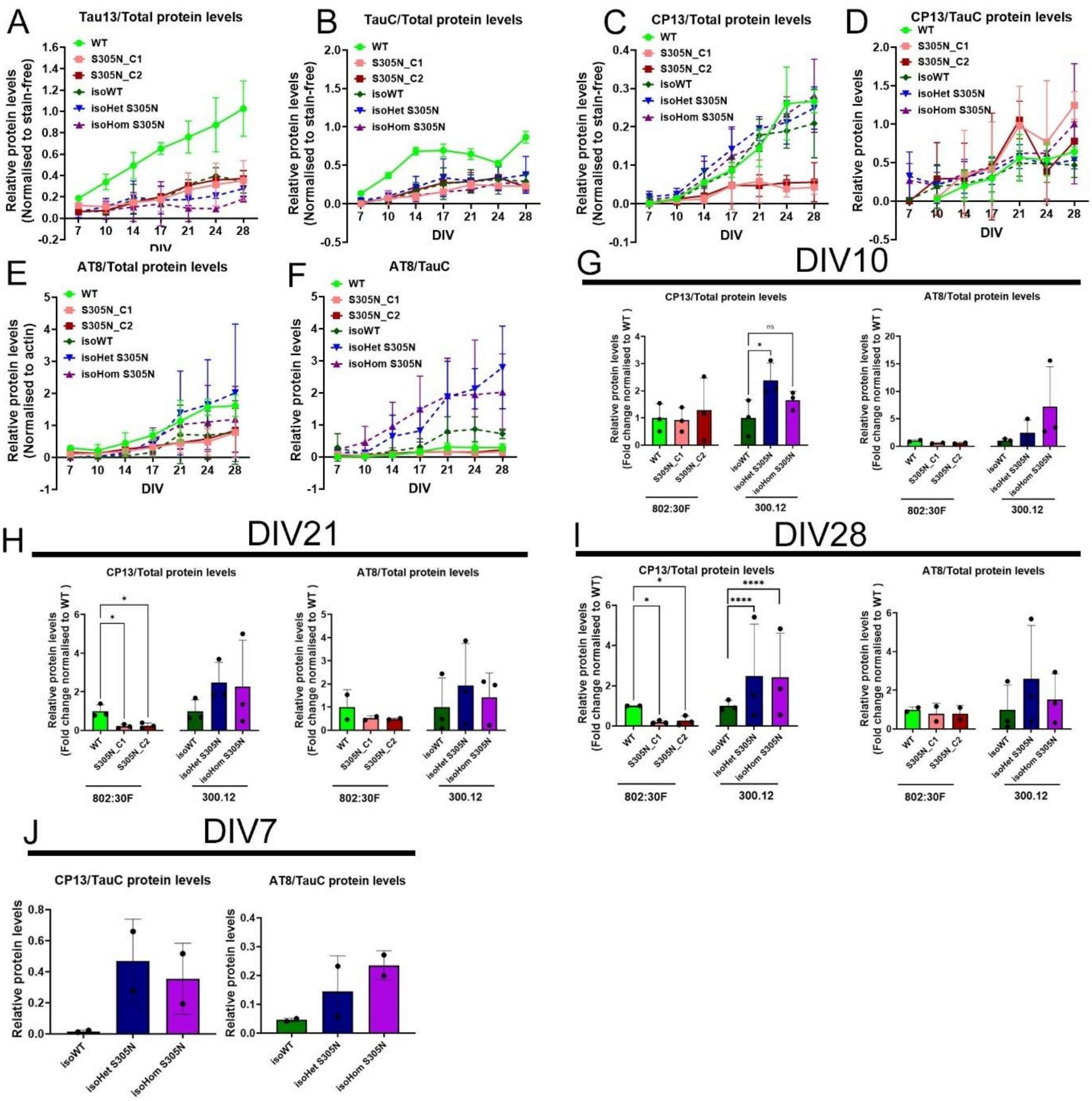

Fig. S5. Expression levels of total and phosphorylated tau, related to Fig 4, over time. (A–B) Protein levels of total tau detected using Tau13 (A) and TauC (B) antibodies, normalized to total protein (stain-free imaging), across all neuronal lines over time. (C–D) Expression of phosphorylated tau detected using CP13, normalized to total protein (C) and to TauC (D), across all neuronal lines. (E–F) Expression of phosphorylated tau detected using AT8, normalized to total protein (E) and to TauC (F), across all neuronal lines. (G–I) Protein levels of CP13 (left) and AT8 (right) at DIV10 (G), DIV21 (H), and DIV28 (I), normalized to total protein (stain-free imaging) in all neuronal lines. (J) Protein levels of CP13 (left) and AT8 (right) normalized to TauC at DIV7 in isoWT, isoHet, and isoHom S305N neurons. Statistical analysis was performed using repeated measures (RM) one-way ANOVA with Dunnett's post-hoc test to compare S305N_C1 and C2 vs WT, and isoHet/isoHom S305N vs isoWT. These tests were selected due to variability in immunoblot replicates. Data are presented as mean ± SD; *, $p<0.05$; ****, $p<0.0001$ n = 2-3 independent differentiations per presented neuronal line.

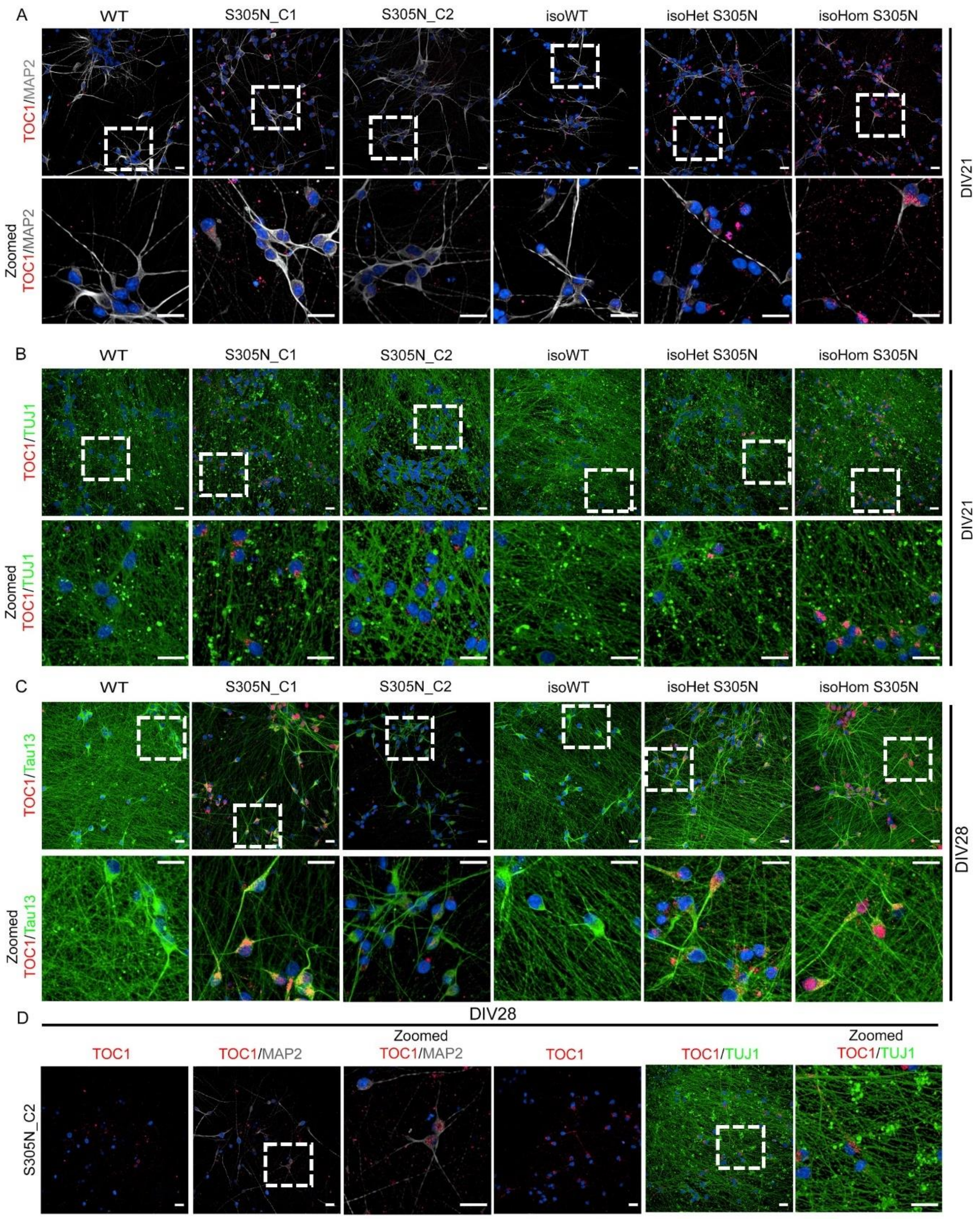

Fig. S6. TOC1 expression at DIV21 and colocalization with Tau13 in S305N i^{3}N neurons related to Fig. 5. (A) Representative images of TOC1 and MAP2 co-labelling at DIV21 for all neuronal lines. (B) Representative images of TOC1 and TUJ1 co-staining at DIV21 for all neuronal lines. (C) Representative images of TOC1 and Tau13 co-staining at DIV28 for all neuronal lines. (D) Representative images of S305N_C2 DIV28 i^{3}N neurons stained with either TOC1 and MAP2 or TOC1 and TUJ1.

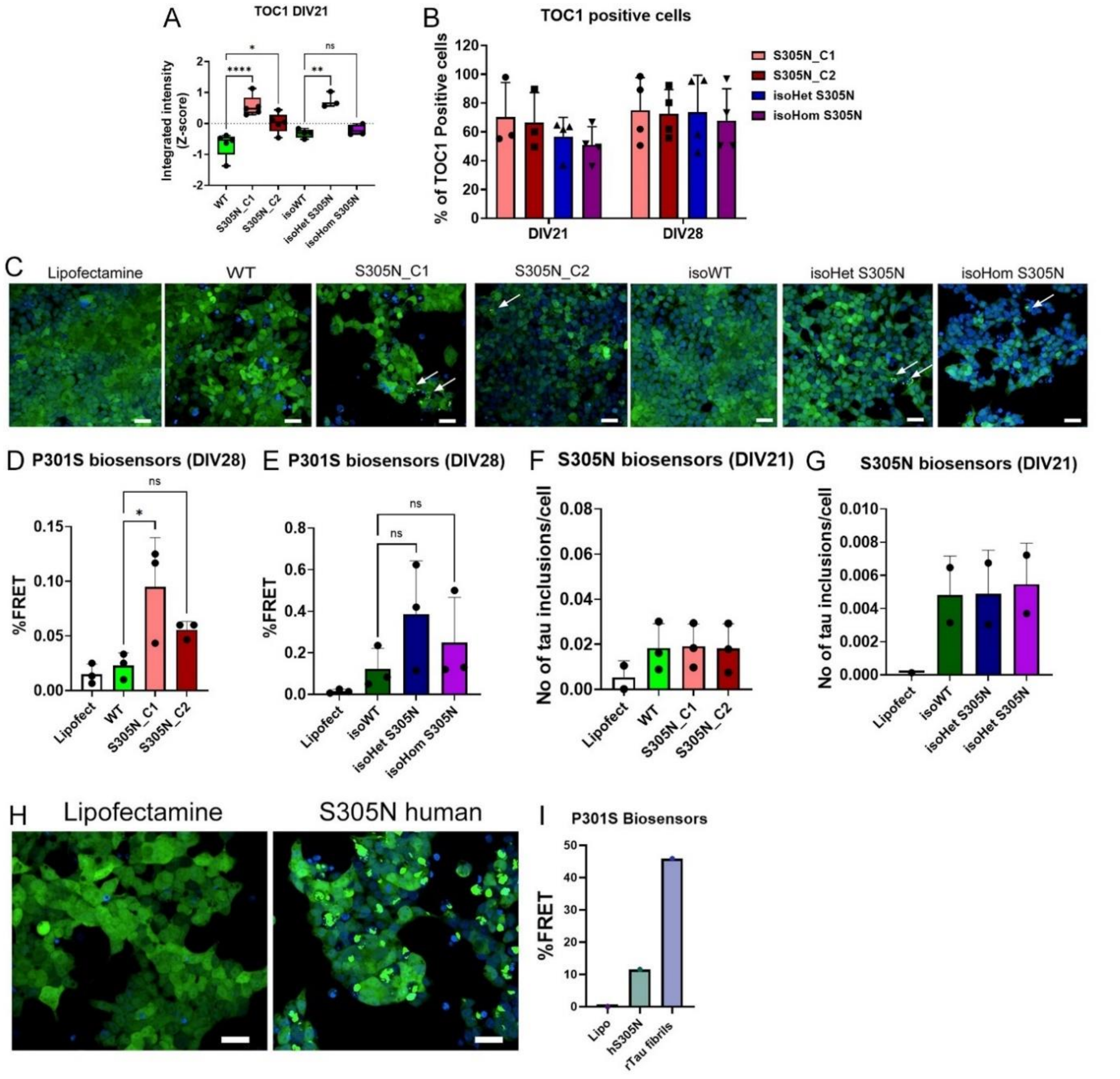

Fig. S7. i^{3}N neurons with a S305N mutation form endogenous seed-competent tau related to Fig. 5. (A) Quantification of TOC1 signal from DIV21 i^{3}N neurons in MAP2-positive area. Statistical analysis was performed using one-way ANOVA followed by Tukey's post-hoc test to compare mutant lines with their respective isogenic controls (mean ± SD, n = 4–5 independent differentiations per presented neuronal line). (B) Percentage of TOC1-positive neurons in i^{3}N cultures with S305N mutations. (C) Representative images showing tau seeding activity in S305N biosensors seeded with DIV28 i^{3}N neuron lysates from all lines. (D–E) Quantification of seeding activity at DIV28 in P301S biosensors. Statistical analysis was performed using one-way ANOVA followed by Tukey's post-hoc test (mean ± SD, n = 3 independent differentiations per presented neuronal line). (F–G) Quantification of seeding activity at DIV21 in S305N biosensors. Statistical analysis was performed using one-way ANOVA followed by Tukey's post-hoc test (mean ± SD, n = 2–3 independent differentiations per presented neuronal line). (H) Representative images showing tau seeding activity in S305N biosensors seeded with human S305N brain tissue. (I) Quantification of seeding activity from 3 µg of frozen human S305N brain tissue using the S305N biosensors. *, $p<0.05$.

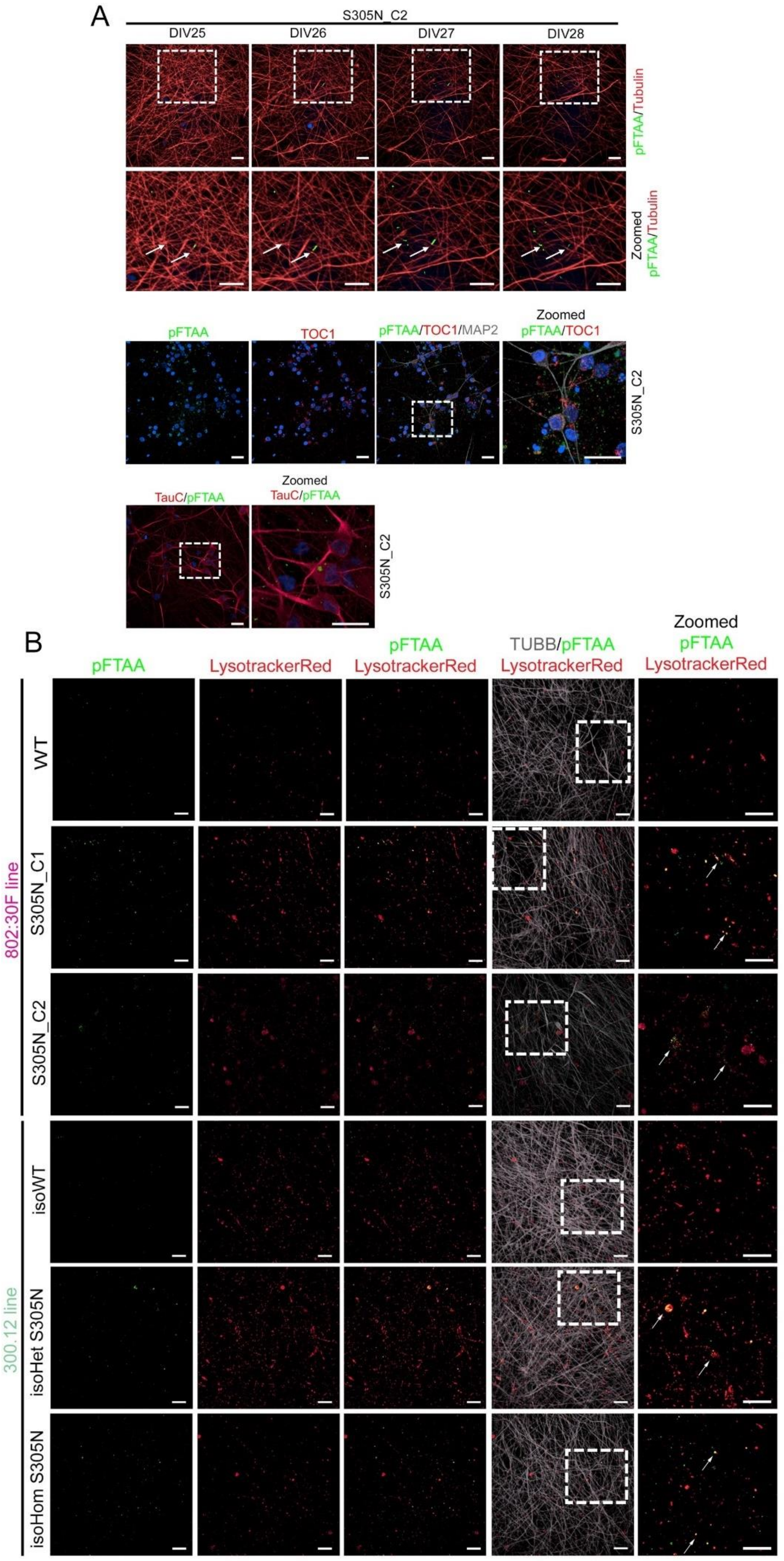
A
S305N_C2
DIV25
DIV26
DIV27
DIV28
pFTAA/Tubulin
Zoomed
pFTAA/Tubulin
pFTAA
TOC1
pFTAA/TOC1/MAP2
Zoomed
pFTAA/TOC1
S305N_C2
TauC/pFTAA
Zoomed
TauC/pFTAA
S305N_C2
B
pFTAA
LysotrackerRed
pFTAA
LysotrackerRed
TUBB/pFTAA
LysotrackerRed
Zoomed
pFTAA
LysotrackerRed
802:30F line
WT
S305N_C1
S305N_C2
300.12 line
isoWT
isoHet S305N
isoHom S305N

Fig. S8. pFTAA colocalizes with LysotrackerRed in i^{3}N neurons, related to Fig. 6. (A) Representative images of S305N_C2 i^{3}N neurons stained live with pFTAA and Tubulin over time (top panel), co-stained with TOC1 and MAP2 (middle panel), and TauC (bottom panel). (B) Representative images of all neuronal lines stained with pFTAA, Lysotracker Red, and Tubulin (TUBB) at DIV28.

Table S1: Summary of changes in 4R tau RNA, total tau protein, phosphorylated tau and hyperphosphorylated tau (pTau/TauC) in all lines with S305N mutations. Data were normalized either to cell number for immunocytochemistry (ICC) or to loading control for Western blot (WB). [1]Protein levels of phosphorylated tau were normalized to loading control, [2]Protein levels of phosphorylated tau were normalized to TauC levels.

	Time	DIV21			DIV28		
	**Cell line**	**S305N_C1/C2**	**isoHet S305N**	**isoHom S305N**	**S305N_C1/C2**	**isoHet S305N**	**isoHom S305N**
mRNA	4R MAPT	90%	47%	67%	90%	54%	72%
**Tau aggregation**	Seeding biosensors	No change	No change	No change	↑↑↑	↑↑	↑
	TOC1 expression (ICC)	↑↑	↑↑	No change	↑↑↑	↑↑↑	↑↑
	pFTAA	NA	NA	NA	↑↑↑	↑↑↑↑	↑↑↑
**Total Tau**	Tau13 expression (ICC)	↓↓↓	No change	No change	↓↓↓	↓	No change (↓ with p=0.08)
	Tau13 expression (WB)	↓↓↓	↓	↓↓	↓↓↓	↓	↓↓
	TauC expression (ICC)	↓↓↓	No change	↓↓	↓↓↓	↓	No change
	TauC expression (WB)	↓↓↓	No change	No change	↓↓↓	No change	No change
**pTau**	AT8 expression (ICC)	↑	↑	↑	No change	↑	↑↑
	AT8 expression (WB)[1]	No change	Variable (↑↑)	Variable (↑)	No change	Variable (↑↑)	Variable (↑)
	CP13 expression (ICC)	↓↓↓	↑	↑↑	↓↓↓	↑↑	↑↑
	CP13 expression (WB)[1]	↓↓↓	Variable (↑↑)	Variable (↑↑)	↓↓↓	Variable (↑↑)	Variable (↑↑)
	AT100 expression (ICC)	↓	No change	↑↑	↑	↑↑	↑
**Hyperphosphorylated tau[2]**	AT8/TauC (ICC)	↑↑↑↑	NA	NA	↑↑↑↑	NA	NA
	AT8/TauC (WB)[2]	No change	Variable (↑↑)	↑↑	No change	↑↑↑↑	↑↑↑
	CP13/TauC (ICC)	No change	NA	NA	Variable (↑↑)	NA	NA
	CP13/TauC (WB)[2]	↑↑	No change (trend ↑)	No change (trend ↑)	↑↑	↑	↑↑
